# Increased pathogenicity of pneumococcal serotype 1 is driven by rapid autolysis and release of pneumolysin

**DOI:** 10.1038/s41467-020-15751-6

**Published:** 2020-04-20

**Authors:** Laura C. Jacques, Stavros Panagiotou, Murielle Baltazar, Madikay Senghore, Shadia Khandaker, Rong Xu, Laura Bricio-Moreno, Marie Yang, Christopher G. Dowson, Dean B. Everett, Daniel R. Neill, Aras Kadioglu

**Affiliations:** 1 Department of Clinical Infection, Microbiology and Immunology, Institute of Infection and Global Health, University of Liverpool, Liverpool, UK; 2 Medical Research Council Unit, The Gambia, Bakau, Gambia; 3 School of Life Sciences, University of Warwick, Warwick, UK; 4 Malawi-Liverpool-Wellcome Trust Clinical Research Programme, University of Malawi, College of Medicine, Blantyre, Malawi

**Keywords:** Bacterial pathogenesis, Bacterial toxins, Bacteriology, Infection

## Abstract

*Streptococcus pneumoniae* serotype 1 is the predominant cause of invasive pneumococcal disease in sub-Saharan Africa, but the mechanism behind its increased invasiveness is not well understood. Here, we use mouse models of lung infection to identify virulence factors associated with severe bacteraemic pneumonia during serotype-1 (ST217) infection. We use BALB/c mice, which are highly resistant to pneumococcal pneumonia when infected with other serotypes. However, we observe 100% mortality and high levels of bacteraemia within 24 hours when BALB/c mice are intranasally infected with ST217. Serotype 1 produces large quantities of pneumolysin, which is rapidly released due to high levels of bacterial autolysis. This leads to substantial levels of cellular cytotoxicity and breakdown of tight junctions between cells, allowing a route for rapid bacterial dissemination from the respiratory tract into the blood. Thus, our results offer an explanation for the increased invasiveness of serotype 1.

## Abstract (web summary)

The mechanisms behind the high invasiveness of *Streptococcus pneumoniae* serotype 1 are unclear. Here, Jacques et al. show that this feature is due to overproduction and rapid release of pneumolysin, which induces cytotoxicity and breakdown of tight junctions, allowing rapid bacterial dissemination from the respiratory tract into the blood.

## Introduction

*Streptococcus pneumoniae* (the pneumococcus) is a major human pathogen responsible for a range of severe diseases including pneumonia, meningitis and bacteraemia^1^. The pneumococcus accounts for significant morbidity and mortality globally, particularly in high risk groups such as the elderly, infants and the immunocompromised^2^.

Serotype 1 has unusual clinical, epidemiological, genetic and microbiological characteristics, making it the most prevalent of the highly invasive pneumococcal serotypes in sub-Saharan Africa^3–6^. Disease rates in the young and in otherwise healthy adults are high, whilst carriage rates are low, even when symptomatic disease is prevalent in the population^3,7^. Epidemiological studies have found that serotype 1 causes a large proportion of the pneumococcal disease seen in HIV-uninfected children, suggesting that serotype 1 is highly virulent and more invasive than other serotypes, enabling it to cause disease in otherwise healthy individuals^7–10^. Despite temporal reductions in disease incidence after the introduction of the Pneumococcal Conjugate Vaccine (PCV13) in 2011, serotype 1 remains the most common serotype causing invasive pneumococcal disease (IPD) in Southern and sub-Saharan Africa^11^.

The pneumococcal toxin pneumolysin is expressed by virtually all clinical isolates and has been described as a key virulence factor contributing to high morbidity and mortality rates in invasive disease^12–16^. Pneumolysin is a member of the cholesterol dependent cytolysin (CDC) family of membrane-binding toxins^17^. It lyses cells with cholesterol in their membranes, activates host complement and induces pro-inflammatory reactions in immune cells^18,19^. For example, experiments in human lung tissue demonstrate that detection of pneumolysin by NLRP3 inflammasomes mediates production of inflammatory cytokines such as IL-1β and IL-8^20^. At lytic concentrations, pneumolysin causes widespread cellular and tissue damage, allowing for increased bacterial replication and tissue invasion^14^. As pneumolysin is a cytosolic toxin, release is mediated by an autolysin-dependant process^21^. This process is characterised by cell wall degradation by a peptidoglycan hydrolase (autolysin), the most common of which is known as LytA^22^. As pneumolysin lacks an N terminal secretion signal sequence, lysis of the bacteria by autolysin is essential for toxin release^21,23,24^. Genetically designed pneumolysin-deficient (PLY-) and autolysin (LytA^−^) deficient mutant pneumococcal strains have been shown to be attenuated in virulence after intranasal administration into mice^12,25–28^. Hence, pneumolysin and autolysin work hand in hand as key pneumococcal virulence factors.

The presence of cytotoxic pneumolysin in the lung during the initial phase of pneumonia contributes to the development of bacteraemia, facilitating penetration of bacteria from the alveoli into the interstitium of the lung, and dissemination of pneumococci into the bloodstream, during experimental models of pneumonia^29,30^.

The importance of pneumolysin in pneumococcal carriage and invasive disease has been well characterised in serotype 2 (D39) infection but little is known about the combinations of key virulence factors that contribute to serotype 1 disease pathogenesis. Post vaccine studies are ongoing to monitor the effect of PCV13 introduction on serotype 1 distribution, however, an indirect cohort analysis from a surveillance programme failed to demonstrate significant protection against serotype 1 IPD by PCV13^31^. In addition, phylogenetic analysis of invasive serotype 1 pneumococcal isolates in South Africa show increases in genetic diversity and an increase of lineages associated with antimicrobial non-susceptibility post-PCV13^11^. Hence, an understanding of the contribution of key virulence factors to pneumococcal pathogenesis could aid both future vaccine design and potential therapeutics for serotype 1 IPD.

To identify factors that make key contributions to serotype 1 disease pathogenesis, we used murine models of experimental pneumococcal pneumonia and nasopharyngeal carriage. Experiments were performed with the pneumococcal pneumonia-resistant BALB/c strain of mice^32–34^, and disease severity of infection with African serotype 1 (ST217 and ST3081) was compared to serotypes 2 (D39), 5, 6B and 7F infection. African serotype 1 clonal complex ST217 was used, as this has been the dominant clone amongst serotype 1 isolates in Africa since 1989^6,11^.

Here, we demonstrate that in a normally resistant BALB/c pneumonia model, African serotype 1 induced 100% mortality, whilst mice challenged with the other serotypes all survived. Serotype 1 virulence was driven by rapid bacterial autolysis, which leads to the release of large quantities of pneumolysin, enabling rapid bacterial dissemination into the bloodstream.

## Results

### Serotype 1 pneumococci display high tissue invasiveness

**Fig. 1:**
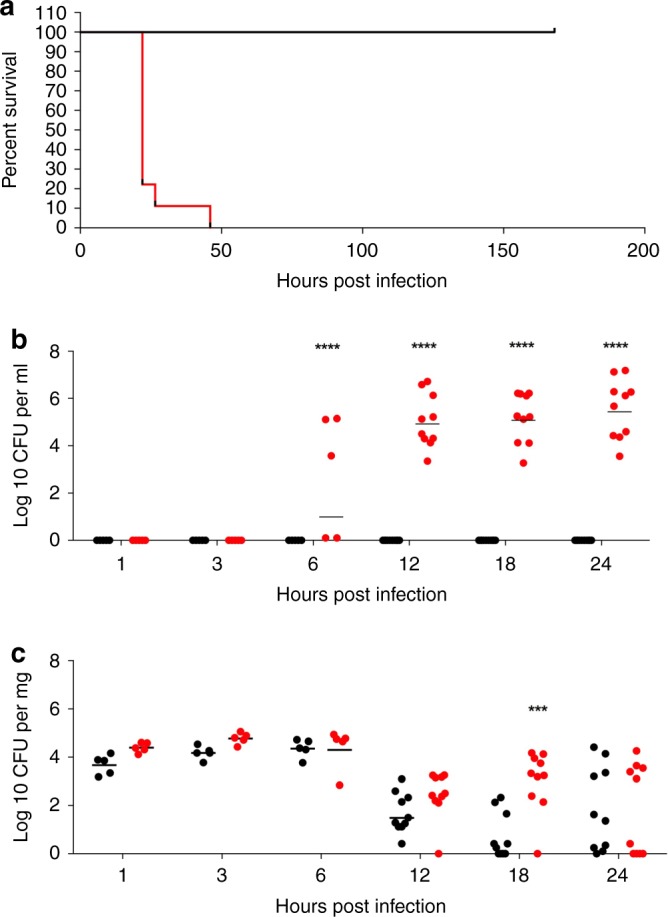
Comparison of Serotype 1 (ST217S) and Serotype 2 (D39) in BALB/c mouse pneumonia model. **a** Kaplan–Meier survival curve showing survival times of mice infected intranasally (IN) with 1 × 10^6^ colony forming units (CFU) of serotype 1 (ST217S) (red line) or serotype 2 (D39) (black line). 1 (ST217S) infected mice *n* = 9 and 2 (D39) infected mice *n* = 10. **b** Blood bacterial load in 1 (ST217S) infected mice (red dots) and 2 (D39) infected mice (black dots) at 1,3, 6, 12, 18 and 24 h post intranasal infection (CFU per ml). **c** Lung bacterial load in 1 (ST217S) infected mice (red dots) and 2 (D39) infected mice (black dots) at 1, 3, 6 12, 18 and 24 h post intranasal infection CFU per mg) (each dot represents one mouse). Data are presented as follows; each symbol represents an individual mouse and geometric mean is shown by horizontal bars. Statistical analysis was performed using Two-way ANOVA and Sidaks’s post-test. ***P-value = 0.0001, ****P-value < 0.0001. Source data are provided as a Source Data file.

BALB/c mice intranasally infected with 10^6^ colony forming units (CFU) of serotype 1 (ST217) displayed 100% mortality within 48 h of infection, compared with 0% mortality in mice infected with the same concentration of serotype 2 (D39) (Fig. 1a).

In addition, as early as 6 h post-infection, 60% of serotype 1 infected mice had significant bacteremia (mean CFU: 5 × 10^4^ cfu per ml), increasing to 100% of mice by 12 h post infection until time of death (mean CFU: 3 × 10^6^ cfu per ml). No bacteria were detectable in the blood of serotype 2 (D39)-infected mice at any time point tested (Fig. 1b). Despite these significant differences in bacteremia, no significant differences in lung bacterial loads were observed between serotype 2 and serotype 1-infected mice over the first 12 h post infection (Fig. 1c) and so we hypothesised that the level of bacterial load in lungs is not directly responsible for bacterial dissemination into the blood stream in this murine model of pneumonia.

**Fig. 2:**
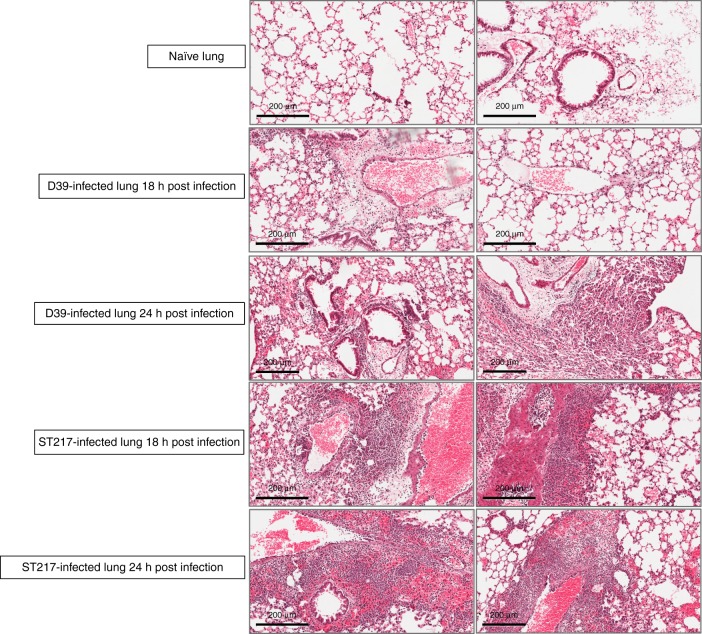
Lung histopathology during murine pneumonia infection with Serotype 1 (ST217) and Serotype 2 (D39) S. pneumoniae. Mice were infected as described in Fig. 1. Lung sections from infected mice were stained with haematoxylin and eosin to show changes in lung pathology during pneumococcal infection with either 1 (ST217S) or 2(D39) at 18 and 24 h post infection (5 mice/group/timepoint). Lung sections from naïve mice (*n* = 5) are also shown. Scale bars =  200 μm.

Lung tissue from naïve and *S. pneumoniae*-infected mice was used to examine damage, via histopathology, during pneumococcal pneumonia (Fig. 2). Substantially greater levels of immune cell infiltration, inflammation and pathology were observed in ST217-infected lungs as compared to D39 at equivalent timepoints. By 18 h post-infection, lungs infected with ST217 exhibited hypertrophy of bronchiole walls, heavy cellular infiltration around bronchioles and evidence of oedema. By 24 h post-infection, extension of inflammatory cell infiltration from bronchioles and perivascular areas into the surrounding lung parenchyma was apparent with several focal areas of heavy consolidation becoming larger and more diffuse. Although a gradual increase in cellular infiltration and inflammation was also observed in D39-infected lungs, this was substantially less prominent than that seen in ST217-infected lungs.

Immune cell analysis of ST217 and D39 infected lungs was performed at 6, 12 and 24 hours post infection (Supplementary Figs. 1A–C, 9 and 10). Mice infected with ST217 had significantly higher numbers of Gr-1^+^ neutrophils in the lungs 6 h post infection compared to D39-infected mice (*P* < 0.01) and this trend continued at 12 and 24 h post-infection, despite equivalent CFU levels across these timepoints in D39 and ST217-infected mice, again suggesting that the drivers of inflammation are not bacterial numbers alone. Interestingly, despite early increases in Foxp3^+^ T regulatory cell numbers in ST217-infected lungs, by 24 h post-infection D39-infected mice had significantly higher numbers of Foxp3^+^ cells.

**Fig. 3:**
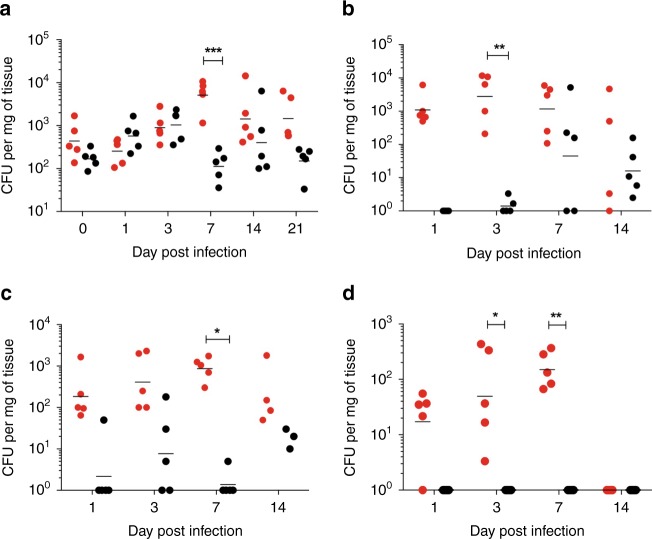
Comparison of Serotype 1 (ST217S) and Serotype 2 (D39) in BALB/c mouse nasopharyngeal carriage model. Mice were infected by intranasal administration with either 10^5^ CFU of 1(ST217S) or 2(D39) and culled at 0, 1, 3, 7, 14 or 21 days post infection. In all, 5–10 mice per group were culled and tissues were taken for analysis of bacterial load. Data presented as follows; each symbol represents an individual mouse and error bars show the geometric mean. Mice infected with 1 (ST217S) are shown by red dots and 2(D39) infected shown as black dots. Statistical analysis was performed using Two-way ANOVA and Sidaks comparison test. **a** CFU counts in the nasopharynx of infected mice. ***P value = 0.0008 (**b**) CFU counts in the Olfactory epithelium of infected mice. **P value = 0.0025. **c** CFU counts in the Olfactory Bulb of infected mice. *P value = 0.0341. **d** CFU counts in the brain of infected mice. *P value = 0.0200, **P value = 0.0070. Source data are provided as a Source Data file.

Dissemination of serotype 1 (ST217) in a non-lethal murine nasopharyngeal carriage model was also investigated (Fig. 3). During a 21-day carriage experiment in BALB/c mice, comparing D39 and ST217 carriage patterns, nasopharyngeal bacterial burdens were comparable on all days except day 7 post infection when a significantly higher density of serotype 1 colonisation was detected (Fig. 3a). This higher density of serotype 1 colonisation prevailed over the next 14 days. Flow cytometry was performed to analyse the fluctuation of immune cell numbers in the nasopharynx over 21 days of colonisation in ST217-infected mice compared to D39-infected (Supplementary Figs. 1d–g, 10 and 11). Both groups of infected mice showed a similar trend of neutrophil (CD45^+^ Gr-1^+^) and macrophage (CD45^+^ CD11b^+^ F4/80^+^) numbers over time. A significant difference in both neutrophil and macrophage numbers was observed at day 14 post infection with D39-infected mice showing higher numbers of macrophages and neutrophils compared to ST217-infected mice. In addition, cells associated with the adaptive immune response were also monitored over 21 days in the nasopharynx. No significant differences in the numbers of Foxp3^+^ T regulatory cells or RORγT^+^ Th17 cells were observed between ST217 and D39 colonisation. In addition, no significant differences were observed in the number of Foxp3^+^ cells expressing TGFβ or Th17 cells expressing IL-17A between infection with the different serotypes.

Of note, serotype 1 was able to invade and colonise the olfactory tissues at a substantially higher density over 14 days than D39, with significantly higher ST217 CFU at days 5 and 7 post infection in olfactory epithelia and bulb respectively (Fig. 3b, c). Importantly, while serotype 1 colonies were detected in the brain during the first 7 days of nasopharyngeal carriage, no D39 colonies were detected throughout this experiment (Fig. 3d), demonstrating the capacity of serotype 1 to translocate across nasopharyngeal to olfactory and ultimately brain tissue.

Neither ST217, nor D39, colonies were detected in the blood at any timepoint (data not shown), suggesting that serotype 1 may utilise host invasion of olfactory tissues to enter the brain, bypassing the blood-brain barrier. Indeed, direct invasion from the nasopharynx to the brain via the olfactory tissues has been previously reported for serotype 19F, in immunocompromised XID mice that are highly susceptible to pneumococcal infection^35^. To the best of our knowledge, this is the first reported description of this pathway of translocation from nasopharyngeal tissues to meninges for *Streptococcus pneumoniae* in immunocompetent mice.

### Comparison of invasive clinical isolates in a pneumonia model

**Fig. 4:**
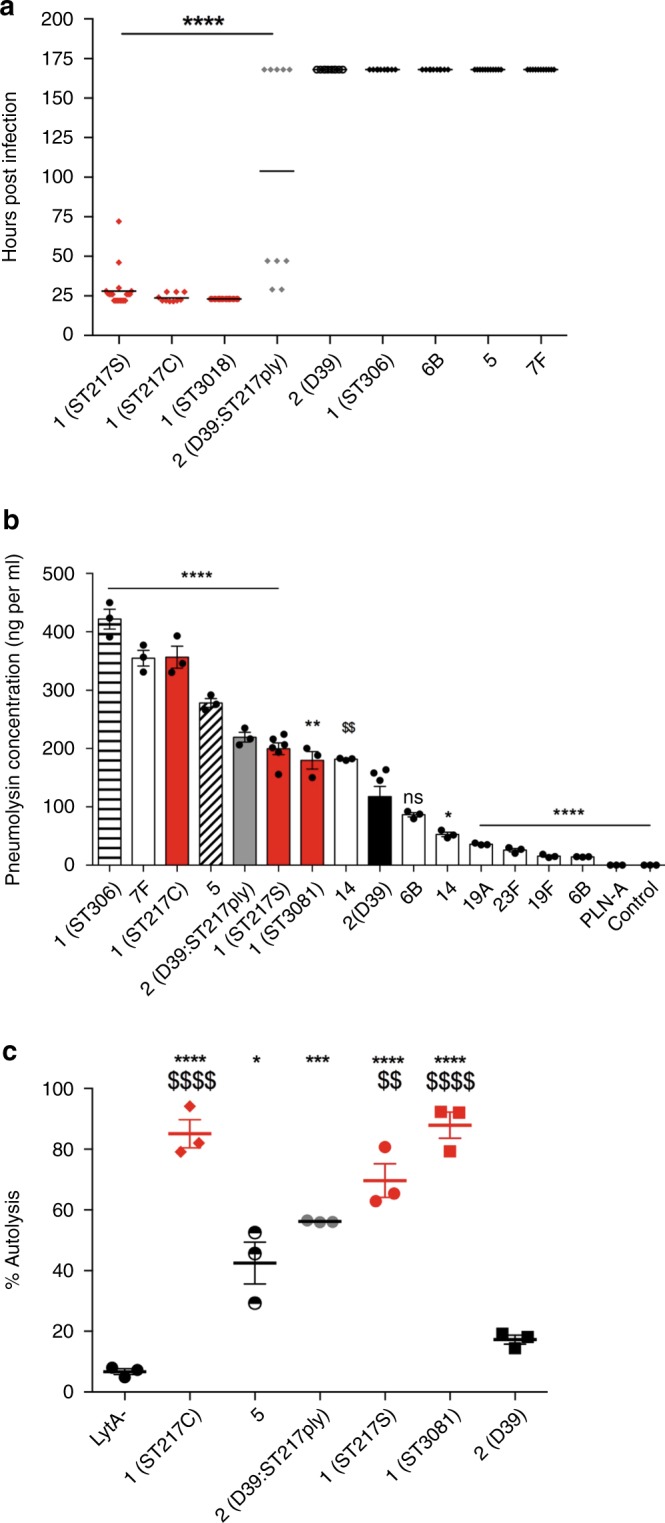
A combination of pneumolysin production and rates of autolysis correlate with pneumococcal virulence. **a** Mice were infected with 1 × 10^6^ colony forming units (CFU*) S. pneumoniae* by intranasal administration and monitored closely for signs of ill-health. If infected mice became lethargic, they were culled, survival times noted and CFU from blood determined by Miles and Misra dilution. Survival times of mice infected with African serotype 1 strains (red) compared to other clinically relevant strains (black) and a D39 strain expressing the ST217S pneumolysin (grey) (10 mice/group). Experiment was ended at 168 h. Statistical analysis was performed using One-way ANOVA and Dunnett’s multiple comparisons test: ****P-value < 0.0001 vs D39. **b** ELISA-based quantification of the amount of pneumolysin released by different strains and serotypes (10^7^ CFU) upon lysis by penicillin/streptomycin treatment. Data represented as mean ± SEM. For all serotypes *n* = 3 biologically independent samples, whereas for 1(ST217S) and 2(D39), *n* = 6 are shown (six biologically independent samples over two independent experiments). Statistical analysis was performed by one-way ANOVA with Dunnett’s multiple comparison test. Asterisk represent significant differences versus serotype 2 (D39). *P < 0.0132, **P < 0.01, ****P < 0.0001, ns = not significant. **c** Bacteria (OD_600_ 1.0) were incubated at 37 °C and 175 rpm with 0.01% Triton X. At 60 min post treatment, OD_600_ was measured and converted to a percentage of the original OD_600_ reading. Percentage autolysis represents the percentage decrease of original OD_600_ reading. Statistical analysis was performed by Two-way ANOVA and Tukey’s multiple comparisons test. Rates of autolysis were measured in triplicates; data are presented as mean ± SEM. Asterisk represent comparisons of serotype 1 to 2(D39) and $ represents comparisons of serotype 1 to serotype 5. (ns = non significant. *P = 0.0103, ^$$^*P* = 0.0056, ***P = 0.0002, ****^/$$$$^*P* < 0.0001). Source data are provided as a Source Data file.

To assess whether high mortality rates in resistant BALB/c mice were unique to serotype 1 (ST217) infection, the pneumonia survival model was repeated with different clinical isolates of *S. pneumoniae* (Fig. 4a). The isolates tested included the invasive clinical serotypes 1, 5, 7F and 6B, all of which are included in the current PCV13 vaccine^36^. Additional serotype 1 isolates were also included in these experiments, including another ST217 isolate from Karonga, Malawi (ST217C), an ST3081 from the Gambia and a European serotype 1 isolate (ST306) expressing a non-haemolytic pneumolysin. Strikingly, while 100% mortality was observed in BALB/c mice infected with all African serotype 1 isolates (ST217S, ST217C and ST3081), 100% survival was seen with all other serotypes tested, including the non-haemolytic serotype 1 strain ST306 (Fig. 4a).

### Comparison of pneumolysin production in clinical isolates

ST306 expresses a non-haemolytic pneumolysin and was the only serotype 1 sequence type not to cause any mortality in vivo, further suggesting that pneumolysin is a key determinant of serotype 1 virulence in vivo. The amount of pneumolysin released upon total lysis of 10^7^ CFU of different pneumococcal serotypes was determined by ELISA (Fig. 4b). Total pneumolysin content of lysed CFU varied considerably between isolates, from 20 ng per ml for serotype 19F to over 400 ng per ml for serotype 1 (ST306) (Fig. 4b). Serotype 1 isolates produced between 200 and 420 ng of pneumolysin upon lysis, a range of production at the upper end of the strains tested, and significantly more than that produced by D39, but comparable with that of the non-lethal serotype 5 and 7F strains.

In order to determine whether pneumolysin was one of the factors responsible for ST217 virulence, we replaced the *ply* gene of D39 with that of ST217. Mice infected with D39:ST217*ply* showed a phenotype intermediate between those infected with D39 and those infected with ST217. We observed 50% mortality in D39:ST217*ply* infected mice (Fig. 4a, Supplementary Fig. 2a) and bacteraemia was observed as early as 3 h post-infection (Supplementary Fig. 2b). However, by 24 h post-infection, only 50% of mice remained bacteraemic (Supplementary Fig. 2b), compared with 100% of ST217-infected animals (Fig. 1b). D39:ST217*ply* expressed levels of pneumolysin that were significantly higher than D39 and comparable to those of ST217 (Fig. 4b), suggesting that virulence of ST217 is at least partially due to high levels of pneumolysin production.

### Significantly higher autolysis rates found in Serotype 1 isolates

The release of pneumolysin depends upon autolysin-dependent lysis of pneumococci^37^. As autolysis is a key determinant of toxin release, measurements of bacterial autolysis were used to assess the amount of pneumolysin released from the cytoplasm of the cells.

Triton-X induced autolysis assays were performed to assess the relative rates of autolysis amongst different pneumococcal isolates with moderate to high levels of pneumolysin production (Fig. 4c and Supplementary Fig. 3). A D39 LytA knockout mutant (LytA^−^) was used as a control in these experiments to show that Triton-X induced autolysis was dependent on LytA activation. OD_600_ readings significantly decreased (shown as percentage increase in autolysis) for serotype 1; ST3081, ST217C, ST217S and D39:ST217*ply* strains 60 min after Triton X treatment compared to serotype 2 (D39). Interestingly, although serotype 5 produced pneumolysin levels comparable with those of serotype 1, it caused no mortality in the BALB/c pneumonia model and rates of Triton-X induced autolysis were found to be significantly lower in serotype 5 compared to serotype 1 isolates after 60 min of Triton X treatment. Consistent with its intermediate virulence phenotype, the D39 strain expressing the ST217 pneumolysin (D39:ST217*ply)* had an autolysis rate that was significantly higher than that of the parental D39 strain but lower than that of the serotype 1 isolates. Serotype 5 and the D39:ST217*ply* strain had initially high autolysis rates but these plateaued between 60 and 120 min post Triton X treatment (Supplementary Fig. 3). These findings suggest that autolysis is significantly more rapid in serotype 1, leading to enhanced release of pneumolysin, as compared to other serotypes.

### Serotype 1 supernatant aids virulence of D39 in pneumonia model

**Fig. 5:**
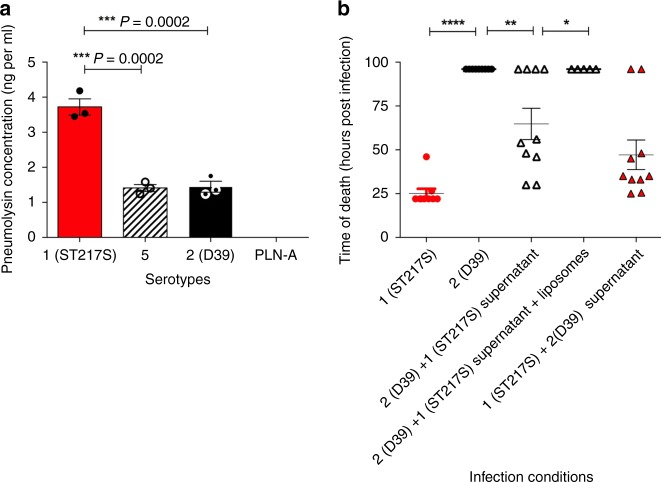
High concentration of pneumolysin in infection dose correlates with high mortality rates in BALB/c mice. **a** In separate tubes, 2 × 10^7^ CFU per ml of 1 (ST217S), 2(D39), serotype 5 and PLN-A were prepared in PBS. Dose preparations were incubated at room temperature for 60 min before a sample of supernatant was removed and pneumolysin detection ELISA used to quantify the amount of pneumolysin released into the supernatant for each strain. Experiments were performed in triplicate and error bars represent mean ± SEM. Statistical analysis was performed using a one-way ANOVA and Tukey's multiple comparison test. **b** Survival times of mice infected with different combinations of 10^6^ CFU of either serotype 1 (ST217S) or serotype 2 (D39) *S. pneumoniae* in 50 μl bacterial dose supernatant that had been incubated at room temperature for 60 min, 10 mice per group. For one group of mice, the supernatant from ST217S was treated with liposomes (a combination of 2 μg/ml Cholesterol:sphingomyelin (66 mol/%cholesterol) and sphingomyelin-only liposomes) for 30 min. Each symbol represents one mouse. Errors bars represent mean ± SEM. Statistical analysis was performed using One-way ANOVA and Tukey’s multiple comparison test: *P-value = 0.0398, **P-value < 0.0093 and ****P-value < 0.0001. Source data are provided as a Source Data file.

To examine whether natural rates of autolysis (not induced by Triton-X) might contribute to in vivo virulence, and to determine whether extracellular pneumolysin within the supernatant of the infection inoculum might impact on disease progression, a supernatant switch experiment was designed. Serotype 1 (ST217S) and serotype 2 (D39) inoculum doses (2 × 10^7^ CFU each) were prepared in 1 ml of PBS. The doses were incubated for 60 min at room temperature. An ELISA-based detection method was used to measure the concentration of pneumolysin released into the inoculum dose during this time period (the supernatant). PLN-A (isogenic pneumolysin-negative mutant of D39) was used as a pneumolysin-negative control. Results demonstrate that the serotype 1 inoculum had significantly higher concentrations of pneumolysin in its supernatant compared to the D39 supernatant (***P = 0.0002; Fig. 5a). To investigate the effect of high concentrations of supernatant pneumolysin in the serotype 1 inoculum dose on induction of disease, the supernatants of serotype 1 and D39 inoculum doses were swapped before infection of the mice. D39 and serotype 1 infection doses were prepared in 1 ml of PBS, incubated at room temperature for 60 min and then immediately prior to infection, bacteria were pelleted by centrifugation and the supernatants of the two doses were swapped. Mice were infected with either D39 bacteria resuspended in supernatant from serotype 1 (ST217S) inoculum dose or serotype 1 (ST217S) bacteria resuspended in supernatant from D39 inoculum dose.

High concentrations of pneumolysin in the supernatant of inoculum doses contributed significantly to mortality in the mouse pneumonia model (Fig. 5b). When mice were infected with serotype 1 bacteria and serotype 1 supernatant, 100% mortality was observed with a mean survival time of 25 h compared to 100% survival in mice infected with D39 bacteria in D39 supernatant. When mice were infected with D39 bacteria with serotype 1 supernatant, survival rates decreased from 100% to 40%, with a mean survival time of 62.5 h, and when mice were infected with serotype 1 bacteria with D39 supernatant, mean survival time increased from 25 h to 48 h and 20% survival rates were observed. Finally, when serotype 1 supernatant was pre-treated with cholesterol-containing liposomes (which we have previously shown to strongly bind and sequester pneumolysin)^38^ prior to resuspension of the D39 pellet and infection of mice, survival rates returned to 100%, confirming that the serotype 1 pneumolysin in the supernatant is responsible for increased mortality in D39-infected mice.

### Cytotoxic effects of serotype 1 pneumolysin on lung epithelial cells

**Fig. 6:**
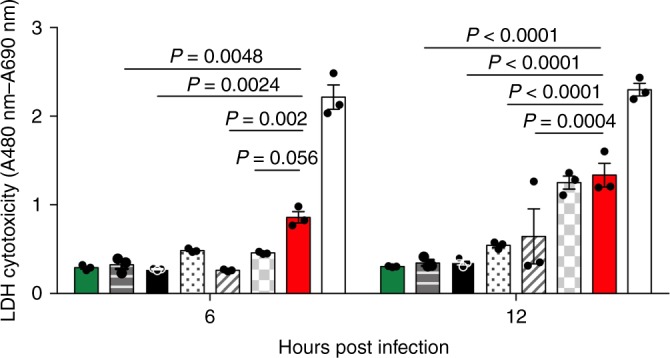
Level of lung epithelial cell cytotoxicity during infection varies with serotype. A549 lung epithelial cells were infected with 6 × 10^5^ CFU *S. pneumoniae* and, at 6 and 12 h post infection, samples of cell culture supernatant were removed and amount of LDH (an indicator of cytotoxic damage) measured. Infections were performed in triplicate wells and error bars represent the mean ± SEM from three independent experiments. Bars, from left to right, are non-infected control cells (green), serotype 1 (ST306) infected (horizontal bars), 2(D39) infected (black), 6B infected (dotted bars), 5 infected (diagonal bars), 7F infected (grey squared bars) and 1(ST217S) infected (red bars). White bars show the LDH positive control. Statistical analysis was performed using Two-way ANOVA and Dunnett’s multiple comparison test. Source data are provided as a Source Data file.

Excess concentrations of pneumolysin in the lung have previously been shown to aid bacterial replication and increase rates of tissue invasion and dissemination into the bloodstream^14,30^. Lactate dehydrogenase (LDH) assays were used to measure cytotoxicity of A549 human lung epithelial cells upon infection with a range of clinical serotypes of *S. pneumoniae* in vitro. Infection with serotype 1 (ST217S) caused significantly more cytotoxic damage to lung epithelial cells compared to other serotypes at equivalent CFU load (Fig. 6). At 6 h post infection, lung epithelial cells cultured with serotype 1 (ST217S) had significantly higher levels of LDH compared to infection with serotype 2 (D39), 5 and 7F. LDH levels were also significantly higher at 12 h post infection with ST217S compared to infection with serotype 5, 6B and 2 (D39).

### Serotype 1 pneumolysin damages epithelial tight junctions

**Fig. 7:**
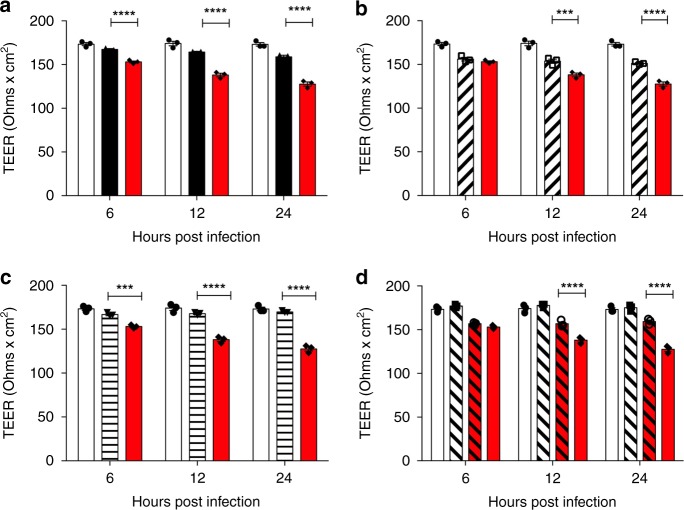
African Serotype 1 causes significant damage to epithelial cell barriers. Human primary alveolar epithelial cells (HPAEpiCs) were cultured on trans-well inserts for 3 days to establish a monolayer. In all, 10^5^ cfu of *S. pneumoniae* were added and trans-epithelial electrical resistance readings (TEER) were taken at 6, 12 and 24 h post infection to assess the damage to tight junctions between epithelial cells. White bars represent control uninfected wells. **a** Comparison between serotype 1 (ST217S) (red bars) and serotype 2 (D39) (black bars) infection. ****P-value < 0.0001. **b** Comparison between 5 (diagonal hashed bars) and serotype 1 (ST217S). ***P-value = 0.0003, ****P-value < 0.0001. **c** Comparison between serotype 1 (ST306) (horizontal striped bars) and serotype 1 (ST217S). ***P-value = 0.0001, ****P-value < 0.0001. **d** Comparison between serotype 1 (ST217S) (red bars) and serotype 1 (ST217S) with the addition of liposomes (red diagonally hashed bars). Clear, diagonally hashed bars represent cells treated with liposomes only. ****P-value < 0.0001. TEER readings were performed in triplicate from duplicate wells from three independent experiments. All statistical analysis was performed using Two-way ANOVA and Tukey’s multiple comparison test. Error bars represents the SEM. Source data are provided as a Source Data file.

Serotype 1 is able to disseminate from the lungs into blood as early as 6 h post infection in vivo (Fig. 1b), leading to systemic infection and high mortality. It also causes early and sustained LDH release from lung epithelial cells in vitro, suggesting that translocation from infected lungs into blood in vivo is most likely due to damage to lung epithelial cell barriers. To further investigate this, in vitro assays were performed to measure the barrier function of human primary pulmonary alveolar cells (Fig. 7) and A549 epithelial cells (Supplementary Fig. 4) during infection with a range of clinical serotypes. A monolayer of cells was infected with *S. pneumoniae*, and trans-epithelial electrical resistance (TEER) measurements taken as an indicator of barrier integrity (Fig. 7). TEER readings are used as an indicator of the permeability of tight junctions between cells grown in culture^39^ and decrease in the TEER value is an indicator of disruption or damage of tight junctions. Infection of both primary and immortalised lung epithelial cells with serotype 1 (ST217) resulted in a significant drop in TEER compared to infection with other isolates of *S. pneumoniae* (D39, 5 and ST306; Fig. 7a–c). The addition of liposomes, to bind and sequester pneumolysin released from ST217, resulted in significantly higher TEER measurements and therefore less damage to the epithelial barrier at 24 h post infection (Fig. 7d).

**Fig. 8:**
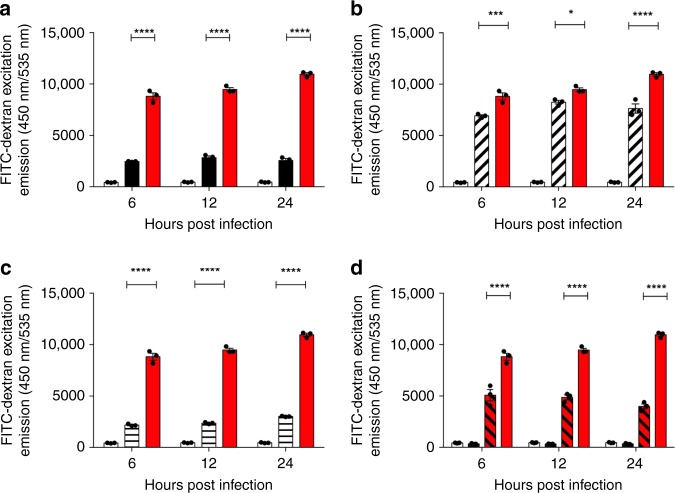
African Serotype 1 causes significant increases to human primary alveolar epithelial cell barrier permeability. Assessment of epithelial cell barrier permeability changes during infection with different *S. pneumoniae* serotypes was performed using fluorescein isothiocyanate (FITC)-dextran (70 kDa, 1 mg per ml), see methods for full details. White bars represent FITC-Dextran emissions from untreated cells. Error bars represent mean ± SEM. Statistical analysis was performed using Two-way ANOVA and Tukey’s multiple comparison test. **a** Comparison of FITC-Dextran excitation emission between serotype 2(D39) (black bars) and 1 (ST217S) (red bars). ****P-value < 0.0001. **b** Comparison of FITC-Dextran excitation emission between serotype 5 (diagonally hashed bars) and 1 (ST217S) (red bars). *P-value = 0.0178, ***P-value = 0.0007, ****P-value < 0.0001. **c** Comparison of FITC-Dextran excitation emission between serotype 1 (ST306) (horizontal bars) and 1 (ST217S) (red bars). ****P-value < 0.0001. **d** Comparison of FITC-Dextran excitation emission between serotype 1 (ST217S) infection with (red, diagonally hashed bars) and without (red bars) the addition of liposomes. ****P-value < 0.0001. As a control, cells were also treated with liposomes in the absence of bacteria (black bars, second column). Source data are provided as a Source Data file.

**Fig. 9:**
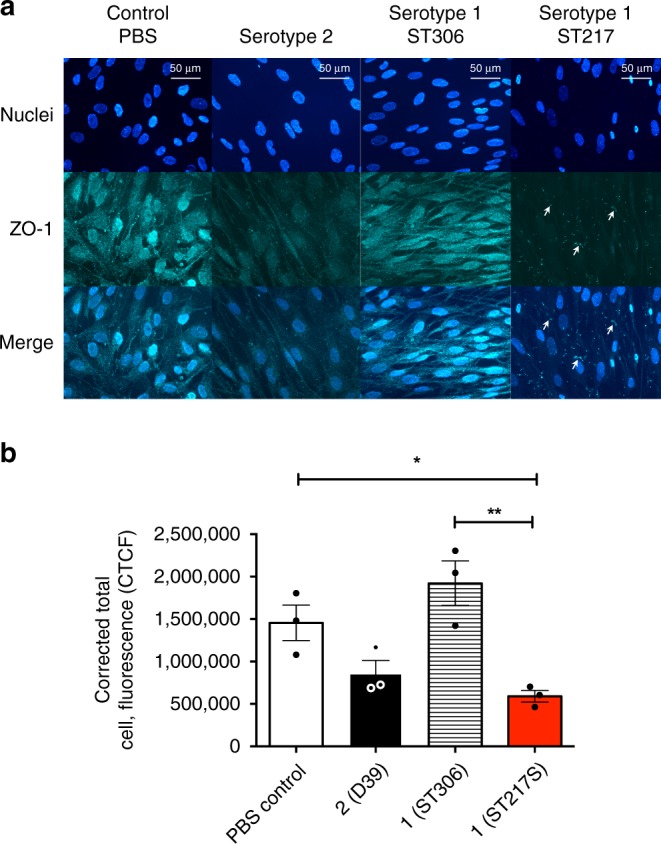
Pneumococcal infection causes degradation of cellular tight junctions. **a** Fluorescent image of Human pulmonary alveolar epithelial cells (HPAEpiC) stained with an antibody against zonula occludens (ZO-1). Cells were co-incubated for 12 h with phosphate buffer saline (PBS), serotype 2 (D39), serotype 1 (sequence type 306) and serotype 1 (sequence type 217**)** at an MOI of bacteria cells – 10:1. Scale = 50 μm. **b** Corrected Total Cell Fluorescence (CTCF) of HPAEpiCs tagged with an antibody against ZO-1. CTCF was calculated by selecting six cells from each merged image. Integrated density, the total area of the cell, and the mean fluorescence of background readings were used to acquire total fluorescence. Results from triplicate experiments shown and error bars represent mean ± SEM. Statisitcal analysis performed using one-way ANOVA and Tukey’s multiple comparison test: *P-value =  0.0460 and **P value = 0.0045. Source data are provided as a Source Data file.

In addition to performing TEER readings on infected HPAEpiCs, the permeability of the tight junctions was assessed using FITC-Dextran (Fig. 8), as well as monitoring the expression of zonula occludens protein 1 (ZO-1) during infection (Fig. 9). Significantly higher emissions of FITC-dextran were observed on the basolateral side of HPAEpiCs infected with serotype 1 (ST217) compared to other serotypes, confirming our findings with TEER readings that suggested that high concentrations of pneumolysin cause significant damage to epithelial cell tight junctions. This effect was significantly reduced in the presence of liposomes. Cell damage by ST217 was independently confirmed using an MTT assay (Supplementary Fig. 5).

ZO-1 protein localizes at tight junction sites in epithelial and endothelial cells and fluorescent staining of this protein can be used to visualise cellular tight junctions^40^. Infection with D39 or ST217 induced markedly decreased abundance in total ZO-1 protein as compared to uninfected and ST306-infected cells (Fig. 9a, b). This effect was most pronounced in ST217-infected cells. There was also evidence of decreased association of ZO-1 with epithelial membranes in ST217-infected cells (Fig. 9a).

## Discussion

*Streptococcus pneumoniae* serotype 1 is the most prevalent and invasive pneumococcal serotype in sub-Saharan Africa, although the bacterial factors driving pathogenesis are not well understood. Here, we used murine infection models and in vitro assays to identify key virulence determinants associated with increased invasiveness of serotype 1.

Previous published work has demonstrated that BALB/c mice are highly resistant to invasive pneumococcal pneumonia when intranasally infected with serotype 2 (D39). No bacterial dissemination from lungs into bloodstream is observed, and bacteria are cleared from the lungs within 3–5 days, resulting in 100% survival rates^32,34,41^. However, our findings show that when BALB/c mice were intranasally infected with African serotype 1 (ST217 and ST3018) isolates, 100% mortality was observed as a result of high levels of bacteremia occurring within 24 h post infection. Indeed, dissemination of serotype 1 from lungs into bloodstream occurred as early as 6 hours post infection with ST217S, demonstrating the highly invasive capacity of serotype 1, even in normally resistant hosts. This invasive phenotype was unique to serotype 1, as infection with other clinical serotypes did not lead to host death. The effect was confirmed to be dependent upon pneumolysin, as a D39 mutant in which the serotype 2 *ply* was replaced with that of serotype 1 ST217 was able to translocate from lungs to blood (just as ST217 did) and cause 50% mortality in BALB/c, as well as expressing levels of pneumolysin that were significantly higher than D39 but comparable to those of ST217.

Sequence comparisons of D39 and ST217 *ply* showed 6 synonymous single-nucleotide polymorphisms (SNPs) in the ST217 pneumolysin gene compared to D39 (Supplementary Fig. 6). Despite these SNPs not leading to amino acid substitutions, mRNA prediction software (RNAfold Web) suggested that the SNPs observed could alter the secondary structure of ST217 *ply* compared to D39 *ply* (Supplementary Fig. 7). These structural differences in ST217 *ply* mRNA may account for the increased levels of pneumolysin produced by ST217 and by the D39 mutant expressing ST217 *ply*.

The initial phase of pneumonia is marked by flooding of the alveoli with bacteria, but very few inflammatory cells^42^. Subsequent breaching of endothelial barriers is thought to facilitate pneumococcal bacteremia and is commonly associated with high mortality rates^43^. This often occurs in the absence of a significant inflammatory response, suggesting that this rapid dissemination is likely to be driven by bacterial toxin-induced damage of the lung epithelial barriers. We demonstrate here that different pneumococcal serotypes produce varying concentrations of pneumolysin at equivalent CFU. However, the amount of pneumolysin produced by serotype 1 was not always significantly greater than that produced by other serotypes, suggesting that it is not simply the amount of pneumolysin produced that determines virulence, but also the rate at which this pneumolysin is released.

Release of pneumolysin is dependent on the activation of autolysin (LytA) which causes rapid cell wall breakdown and cell lysis^22,27,28^. Hence, autolysis is key to enabling release of haemolytic pneumolysin to quickly compromise host defences before a robust immune response can be generated. Indeed, autolysin (LytA-) deficient pneumococcal strains have been shown to be less virulent after intranasal administration into mice^12,27,28^. However, an often overlooked factor is the role played by the rate of autolysis in determining disease progression. A case in point is serotype 5, which produces a high quantity of toxin, but which does not cause mortality or disseminate into the bloodstream during pneumonia in BALB/c mice. Notably, autolysis is significantly slower in serotype 5 than in African serotype 1 isolates, suggesting that pneumolysin release by serotype 5 may not be rapid enough to avoid detection or overcome host defenses in vivo. Although the D39 strain expressing ST217 pneumolysin (D39:ST217*ply*) showed initially high rates of autolysis compared to the D39 parental strain, this effect did not last beyond 120 minutes post Triton-X treatment. Hence, despite D39:ST217*ply* exhibiting elevated autolysis rates relative to D39 (albeit transient), overall, it had reduced autolysis rates relative to other serotype 1 isolates, which may explain why the D39:ST217*ply* strain showed an intermediate virulence phenotype in vivo.

This is supported by observations made in vitro, when different bacterial cultures were incubated with both A549 lung epithelial cells and primary human pulmonary alveolar epithelial cells. When comparing A549 cell infection with serotype 1 (ST217) to other clinical serotypes, significantly higher levels of epithelial cell cytotoxicity (quantified by supernatant LDH levels) was observed as early as 6 h post infection, suggesting that rapid release of pneumolysin by African serotype 1 causes high cellular cytotoxicity to lung epithelial cells in the context of pneumonia infection. In addition, compared to other serotypes tested, ST217 caused significant damage to human primary lung alveolar epithelial cell barriers, as measured using trans-epithelial electrical resistance readings (TEER) and FITC-Dextran permeability assays. This damage was significantly reduced by 24 h post infection with the addition of liposomes, which bind to and sequester pneumolysin. Significantly greater damage to epithelial cell barriers and increases in cellular cytotoxicity by pneumolysin released by serotype 1 isolates are likely to be the main causes of bacterial dissemination observed from lungs into blood and from nasopharynx to brain, in murine models of infection.

Additionally, ZO-1 protein was significantly reduced when lung epithelial cells were infected with ST217 or D39, but not ST306, confirming the impact of haemolytic-pneumolysin expressing serotypes on epithelial tight junctions. ZO-1 is part of a zonula occludens complex (ZO-1, ZO-2, ZO-3), and it is the first to be expressed during the formation of intercellular tight junctions^44^. The loss of ZO-1 was most pronounced in ST217-infected cells, in line with our previous findings that this sequence type rapidly releases pneumolysin. The breakdown of this anchor protein leads to the dissociation of the cell actin cytoskeleton, which may provide a route for paracellular invasion of epithelial cells by ST217, hence aiding its invasiveness.

**Fig. 10:**
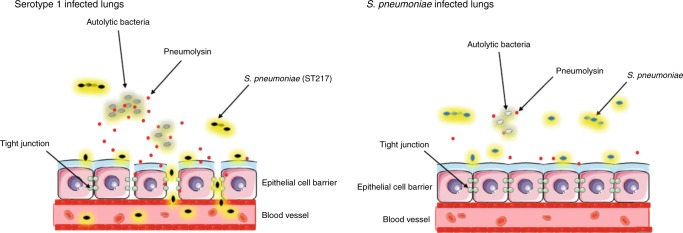
Comparison of pneumococcal pneumonia in serotype 1 compared to serotype 2 infection. In a serotype 1 (ST217/ST3081) infected lung, levels of bacterial lysis are high, resulting in release of high concentrations of the toxin pneumolysin. These high levels of pneumolysin cause damage to tight junctions between epithelial cells and levels of cell cytotoxicity are high. Subsequent damage to lung epithelial cell barriers allows for serotype 1 pneumococci to disseminate from the lungs into the bloodstream and cause bacteraemia. In pneumonia caused by other *S. pneumoniae* serotypes such as 2 (D39), rates of bacterial lysis are lower and therefore lower concentrations of pneumolysin are released into the lung. This results in lower cell cytotoxicity and reduced damage to cell barriers between lungs and blood, allowing for containment of bacteria within the lungs and therefore no development of bacteraemia.

Based on our findings, we propose that the relationship between the amount of toxin produced and its rate of autolysis-dependent release is a key determinant of invasiveness. The combination of these factors drive and control serotype 1 virulence. The proposed mechanism of virulence is explained in summary Fig. 10. Upon entry into the lungs, serotype 1 is likely to undergo rapid autolysis. Increased rates of autolysis will result in rapid release of high concentrations of pneumolysin, which are toxic to lung epithelial cells and cause breakdowns in tight junctions between cells. Increased cellular cytotoxicity and cell permeability allows for bacterial dissemination from lungs into the blood stream where disease progresses to bacteraemia and results in high mortality levels.

The factors governing in vivo autolysis rates largely remain elusive, but may include contributions from the extra-host environment. Indeed, the contribution of environmental factors to African serotype 1 autolysis was highlighted in a recent study, which found that increases in temperature dramatically increased rates of pneumococcal autolysis, leading to increased release of pneumolysin and higher attack rates (invasiveness) of pneumococci colonising the nasopharynx^45^. Given the inherently high rates of autolysis described here for African serotype 1, its sensitivity to higher temperatures may make a significant contribution to invasive serotype 1 disease in sub-Saharan Africa. Epidemiological studies of countries in the African meningitis belt have shown that *S. pneumoniae* serotype 1 causes up to 79% of meningitis cases among older children and working age adults^46^. Data from our murine nasopharyngeal carriage studies demonstrate that serotype 1 disseminates into olfactory tissue and subsequently brain tissue during asymptomatic carriage; an event not observed during serotype 2 colonisation. During infection, rapid autolysis and release of pneumolysin by serotype 1 may enable bacterial dissemination from the nasopharyngeal tissues to the brain via the olfactory bulb. This phenomenon may contribute to the high burden of serotype 1 pneumococcal meningitis in the African meningitis belt.

The potency of free/released pneumolysin in supernatants taken from serotype 1 ST217 inoculum doses was clearly demonstrated when these supernatants were used to drive increased invasiveness of serotype 2 (D39) in a mouse pneumonia model. Addition of ST217 supernatant was sufficient to increase mortality in D39 infection from 0 to 60%. These results demonstrate that serotype 1 pneumolysin is key in driving bacterial dissemination from the lungs into blood, resulting in high mortality rates. Furthermore, D39, which produces lower quantities of pneumolysin, is capable of significantly increased virulence and proliferation in blood if aided across the lung epithelial barrier by serotype 1 pneumolysin, suggesting that penetration of the lung epithelial barrier is the key step in the progression to bacteremia and lethality. Enhanced virulence of D39 when combined with ST217 supernatant was completely reversible with the addition of liposomes that bound and neutralised pneumolysin^38^. The results from these supernatant swap experiments suggest that therapies that target pneumolysin may have significant therapeutic potential in cases of severe pneumococcal pneumonia, where risk of bacterial dissemination into the blood is high. Induction of neutralising anti-toxin antibodies via vaccination with a pneumolysin- or toxoid-containing vaccine may also prevent the key tissue-invasive steps that lead to severe manifestations of disease^47,48^.

The results presented here offer insight into why serotype 1 isolates have a high propensity to cause invasive disease. Our findings demonstrate the key relationship between the amount of toxin produced and its release rate as a determinant of serotype 1 invasiveness and pathogenicity.

Our findings also have implications for our overall understanding of pneumococcal disease pathogenesis, whereby high attack rates may in part result from production of large quantities of pneumolysin, quickly released by rapid autolysis. This highlights the significance of pneumolysin for consideration in future vaccine design and as a key target for new adjunctive therapies.

## Methods

### Ethics statement

This study was performed in strict accordance with UK Home Office guidelines. Animal experiments were performed at the University of Liverpool and were approved by the local animal welfare and ethics committee.

### Mice

For all experiments, 6–10-week-old female BALB/c mice were purchased from Harlan Laboratories (Bicester, UK), and allowed to acclimatise for 7 days prior to use. The animals were housed in the animal facilities under the following conditions; temperature was 21–23 °C and humidity set at 55–65%. Mice were placed in individually ventilated cages (IVC) from Technoplast (GM500). Automatic watering provided reverse osmosis water sterilised by UV radiation and enrichment included nesting material, balcony, dome home and handling tunnel.

### Bacteria

Pneumococci were cultured on blood agar base containing 5% vol/vol horse blood overnight at 37 °C 5% CO_2._. Single colonies of pneumococci were isolated and cultured in brain heart infusion broth (Oxoid, UK) containing 20% (vol/vol) fetal bovine serum until required optical density was reached^27^. See Supplementary Table 2 for details of all *S. pneumoniae* serotypes used in this study.

### Construction of pneumococcal D39:ST217*ply* mutant

Generation of competent *S*. *pneumoniae* D39 cells and subsequent transformation were performed using complete transformation medium (C + Y media, pH = 6.8) method^49^. D39 expressing serotype 1 pneumolysin was constructed by replacing the wild-type D39-*ply* open reading frame with a cassette composed of the serotype 1-*ply* gene followed by the *aphA3* gene (conferring kanamycin resistance) and subsequent selection of kanamycin-resistant transformants (D39:ST217*ply*-*aphA3*) on blood agar base medium supplemented with kanamycin. Transformants were verified by Sanger sequencing. The primers used for mutant construction were: Ply-up-for gatgagcgcgacccagtgccag, Ply-up-rev cagatatccgcagagagatcatcgc, Ply-down-for tggatcctgcttgagtttatctcttgcctagcg, Ply-down-rev gggcttgtttagcacggtcgataac, aphA3-for ctctgcggatatctgtcgctagtattaaatgc, aphA3-rev tcaagcaggatccatcgatac.

### Preparation of challenge dose

Aliquots of *S. pneumoniae* were stored at −70 °C. When required, suspension was thawed at room temperature and bacteria were harvested by centrifugation before being resuspended in sterile phosphate-buffered saline (PBS).

### Infection of mice

Animals were anesthetised with a mixture of O_2_ and Isofluorane and infected intranasally with 1 × 10^6^ CFU *S. pneumoniae* in 50 μl of PBS as described previously^27^. For nasopharyngeal carriage models, inoculation dose was reduced to 1 × 10^5^ CFU in 10 μl of PBS^50^. Mice were periodically scored for clinical signs of disease and culled when they became moderately lethargic or else at pre-determined times post infection. Signs of disease were based on the scheme of Morton^51^. Mice were culled by cervical dislocation and lungs were removed and prepared for assessment of bacterial colony-forming units. Blood samples were taken from tail bleeds or cardiac puncture under terminal anaesthesia.

### Determination of bacterial numbers in nasopharynx, lung and blood

Viable counts of bacteria in lung, nasopharynx, olfactory tissues, brain and blood samples were determined by serial dilution in sterile 1x PBS and plating on blood agar containing 5% (v/v) defibinated horse blood and 40 μg per ml of Gentamycin (Sigma). Plates were incubated overnight at 37 °C 5% (v/v) C0_2_ and bacterial colony numbers were assessed the following day.

### Tissue preparation (murine lungs)

Lung tissue was harvested, weighed, placed into a petri dish and then cut into smaller pieces using a scalpel blade. To help release immune cells via enzymatic digestion, lung tissue was placed in 1.5 ml Eppendorf tubes containing 1 ml of PBS and 10 mg per ml of Collagenase D (Roche). The Eppendorf tubes were then incubated at 37 °C for 30 min. After digestion, tissue was passed through a 40-μm cell strainer (BD Biosciences) and washed with sterile PBS to create a single-cell suspension. Cell suspensions were then centrifuged at 400 × *g* for 5 min. The cell pellet was resuspended in 1x Red blood cell lysis buffer (Sigma) to lyse all red blood cells. The cell suspensions were then centrifuged at 400 × *g* for 5 min and cell pellet resuspended in cryopreservation media, for storage at −80 °C. When needed, aliquots of cells were thawed quickly in the water bath.

### Tissue preparation (murine nasopharynx)

Nasopharyngeal tissue was harvested and placed into bijou tubes containing 3 ml of sterile PBS. The tissue was then mechanically disrupted for ~1 min using a homogenizer (IKA T10). The homogenised tissue was then passed through a 40-μm pore cell strainer and centrifuged at 400 × *g* for 5 min. The cell pellet was either resuspended in cryopreservation media or used for flow cytometry analysis on the same day.

### Flow cytometry

Nasopharynx and lung tissue were collected and prepared as described above. For staining and acquisition, samples were either thawed or used fresh from dissection. Cells were incubated with a 1 in 200 dilution of purified anti- CD16/CD32 Fc blocking antibody (eBiosciences) for 30 min at room temperature. Following incubation with blocking antibody, cell surface markers were stained for. An intracellular monoclonal antibody panel was used to detect both intracellular cytokines and transcription factors for different CD4^+^ T cell subsets. The samples were acquired using a BD FACSCanto flow cytometer (BD Biosciences). See supplementary Table 3 for details of antibody panels used. Supplementary Figs. 9–11 show the gating strategies used to idenitify immune cells of interest.

### Autolysis assay

Triton X-100 induced autolysis assays were performed in triplicate as previously described, with the following modifications^52^. Cultures of *S. pneumoniae* were subcultured into 20 ml of fresh BHI broth and incubated at 37 °C and 200 rpm until an approximate *A*_*600*_ of one was obtained. Cultures were centrifuged for 10 min at maximum speed. Pellets were resuspended in 1 ml of fresh PBS and the *A*_*600*_ was adjusted to 1 in 1 ml of PBS containing 0.01% Triton X-100 (Sigma) in a cuvette. Cuvettes were covered in parafilm and samples vortexed for 10 s and the *A*_*600*_ of the culture at time zero was recorded. Cultures were then incubated at 37 °C and 200 rpm for 3 h and the *A*_*600*_ was measured every 15 min. Triton X-100 induced autolysis was presented as a percentage of the initial *A*_*600*_ at time zero.

### In vitro lung epithelial cell infection

Human lung adenocarcinoma epithelial cell line, A549 (Sigma) and human primary pulmonary alveolar epithelial cells (acquired from ScienCell Research Laboratories – Cat. No 3200) were used to assess cellular damage, disruption to epithelial cell tight junctions and cytokine responses to infection with *S. pneumoniae*. All cells were tested for mycoplasma contamination prior to starting experiments. A549 cells were maintained in DMEM supplemented with 10% foetal bovine serum whilst Alveolar Epithelial Cell Medium (AECM) was used for human primary pulmonary alveolar epithelial cells. In all, 500 μl of cells at 2 × 10^5^ cells per ml density were seeded onto Greiner Thincerts (surface area 0.94 cm^2^, 3 μm pore diameter). Inserts were placed in 12-well tissue culture plates (Greiner) and incubated in 1.5 ml of appropriate cell culture media at 37 °C, 5% CO_2_ for 2 days. Frozen bacterial stocks were thawed, and suspended to a desired final concentration in DMEM + 10% FBS or AECM before addition to tissue culture inserts.

### Trans Epithelial Electrical Resistance measurement

Trans Epithelial Electrical Resistance measurement (TEER) was measured in Ohms using an EVOM2 Epithelial Voltohmmeter (WPI). Wells without cells were used as blank standard. Measurements were done in triplicate and the mean was calculated and blank subtracted. Blank corrected resistance values were multiplied by 0.94 (the surface area of insert) give TEER in Ohms × cm^2^.

### Immunocytochemistry

Briefly, coverslips containing human primary pulmonary alveolar epithelial cells (HPAEpiCs) were washed twice with phosphate-buffered saline (PBS, pH 7.6) and fixed with fresh high-grade 4% paraformaldehyde (PFA) for 10 min. The PFA was aspirated and the coverslips were washed 4 times in PBS for 5 min each time. The coverslips were blocked with 1.5% Normal Goat Serum (NGS, Sigma Aldrich UK) for 2 h at room temperature. Primary antibody incubation was carried out for 2 h at room temperature or overnight at 4 °C with gentle agitation. HPAEpiCs were identified by using a Zonula Occludens primary antibody (Rb polyclonal anti ZO-1 – Abcam ab96587). ZO-1 antibody was used in a 1:100 dilution and the samples were washed in PBS for 5 × 5 min. The corresponding secondary antibody (goat anti-rabbit IgG Abcam ab96883) conjugated to Dylight^®^ (1:1000) was added to the cells for 2 h at room temperature with gentle agitation. Cells were washed in PBS for 5 × 5 min, and the coverslips were inverted onto a drop of mounting medium containing DAPI (Fluoroshield^TM^ Sigma Aldrich UK) on a microscope slide, and stored at 4 °C. The immunostained cells were viewed under fluorescence (Nikon Eclipse 80i fluorescent microscope) with the appropriate excitations for each fluorophore. All images were analysed using ImageJ-Win64.

### Measuring cell fluorescence

To determine the level of cellular fluorescence using microscopy, ImageJ-Win64 software was used as described previously^53^. In brief, an outline was drawn around each cell to measure the area and mean fluorescence, along with several adjacent background readings. The corrected total cell fluorescence was calculated as follows:

$$\left( {{\mathrm{CTCF}}} \right) = \,	{\mathrm{integrated}}\,{\mathrm{density}}\\ 	-\left( {{\mathrm{area}}\,{\mathrm{of}}\,{\mathrm{selected}}\,{\mathrm{cells}}\,\times\,{\mathrm{mean}}\,{\mathrm{fluorescence}}\,{\mathrm{of}}\,{\mathrm{background}}\,{\mathrm{readings}}} \right)$$

### Alveolar permeability assays

Transwell ThinCerts^TM^ (pore size 3.0 μm, 8.4 mm diameter, polyester membrane, Greiner Bio-One, Stonehouse, UK) were coated with Poly-d-Lysine Hydrobromide (Sigma Aldrich, UK). The lower compartments of the Transwell chambers were filled with 1.5 ml Alveolar Epithilial Cell Medium (AECM). HPAEpiCs in 500 μl AECM (5 × 10^4^ cells per well) were seeded on the Thincert’s membrane and reached confluency 24 h post seeding. Cells were coincubated with the appropriate pneumococcal serotype for 6, 12 and 24 h before fluorescein isothiocyanate (FITC)-dextran (70 kDa, 1 mg/ml; Sigma-Aldrich, Irvine, UK) was added to the apical side. In all, 100 μl samples were collected at 0 and 60 min after the FITC addition from the top and the lower compartment. Our Thincert setup was gently agitated for the duration of the permeability assay. Fluorescence (ex: 485 nm; em: 535 nm) was measured using a BMG Omega fluorescent plate reader. The mean fluorescence recorded from the lower compartment of naive cells at the collection point of FITC (60 min) was given the value of 1. Changes to the experimental Thincerts is compared against the control FITC levels. The absolute permeability P [cm per s] was calculated by the following equation^54^:

1 $${\rm{P}} = [{\rm{C}}_{{\rm{t}}}-C_{{\rm{t0}}}]\times {\rm{V/A}} \, \times \, {\rm{t}} \, \times \, {\rm{C}}_{0}$$

*C*(t) is the FITC solution found on the lower compartment (μg per ml), 60 min after the solution was added to the Thincerts, *C*(t_0_) is the concentration of FITC solution at 0 min, *t* is the duration of the flux (seconds), *V* is the volume of the solution found in the lower compartment (cm^3^), *A* is the surface of the Thincert membrane (33 mm^2^) and *C*_0_ is the original concentration of the FITC solution added on the apical side (μg per ml). FITC concentrations were calculated in accordance to a FITC-dextran standard curve.

### Cell viability assays

Prior to the assay, 96-well flat-bottomed micro-plates were coated with 15% Poly-d Lysine Hydrobromide solution, and seeded with 5 × 10^4^ cells per well (total cell media volume of 200 μl per well). The micro-plates were incubated at 37 °C/5% CO_2_ for 24 h. The supernatant was carefully removed and the cells were washed with 100 μl 1xPBS.

### 3-(4,5-Dimethylthiazol-2-yl)-2,5-diphenyltetrazolium bromide assay

The MTT assay measures the reduction of MTT (3-(4,5-Dimethylthiazol-2-yl)-2,5- diphenyltetrazolium bromide) to formazan within living cells. MTT solution was prepared by the addition of 1 mg MTT (Sigma Aldrich®) to 1 ml of phenol red-free media (Gibco®).

Cell culture media was removed and wells were washed with 100 μl PBS followed by the addition of 100 μl MTT (1 mg per ml) per well protected from light. The plate supplemented with MTT was incubated at 37 °C/5% CO_2_ for 4 h. Following incubation, light microscopy was used to observe whether formazan crystals had formed within the cells. Then the supernatant was removed by aspiration and 100 μl of isopropanol was added to the wells to dissolve the crystals formed. Colour change was assessed by absorbance at 560 nm using a ThermoScientific Multiskan plate-reader with the SkanIt (Research Edition) for Multiskan Spectrum 2.2 software.

### Liposomes

Cholesterol and sphingomyelin liposomes were obtained from egg yolk (Sigma Aldrich). Lipids were individually dissolved in chloroform at 1 mg per ml concentrations. For preparation of liposomes, individual lipids were mixed to form Cholesterol:sphingomyelin (66 mol/%cholesterol) and sphingomyelin-only liposomes. Chloroform was completely evaporated with nitrogen gas for 30 min, followed by hydration with PBS (ThermoFisher Scientific). Following incubation for 30 min at 45 °C in an Eppendorf thermomixer with vortexing, liposome preparations were sonicated for 30 min at 4 °C. Final concentration of liposomes was 2 mg/ml.

### Pneumolysin ELISA

ELISAs were used to detect the amount of pneumolysin produced by different serotypes of *S. pneumoniae* (10^7^ CFU per ml) when bacteria were lysed with penicillin/streptomycin antibiotics over the course of an hour at room temperature. Bacterial CFU counts were performed to check full lysis with antibiotics had occurred. In addition, all isolates were checked for penicillin resistance (Supplementary Fig. 8) The ELISA was also used to detect the amount of pneumolysin released into the supernatant when 10^7^ CFU per ml of *S. pneumoniae* when cultured in PBS for 45 min. ELISA plates were coated with 1 μg per well of IgG1-Ply4 (Abcam 71810) and incubated overnight at 4 °C before washing and blocking with Peprotech Blocking Buffer (1% BSA in PBS). Purified recombinant Ply (allele 1, D39) of various concentrations was included as the standard curve in the assay (2-fold dilutions from 400 ng/ml to 3.125 ng/ml), which was incubated for 1 h at 37 °C. After samples were added and incubated, 1 µg per ml of detection antibody (Rabbit polyclonal to PLY antibody, Abcam ab71811) was added to the wells and incubated for 2 h at room temperature. Goat anti-rabbit IgG alkaline phosphatase (Abcam Ab97048) (1:5000 dilution) was added to wells for 30 min at room temperature. After washing, the colour reagent Alkaline Phosphatase yellow pNNP (Sigma p7998) was added and plate incubated for 30 min in the dark. The amount of pneumolysin present was assessed by measuring the plate at 405 nm with an ELISA plate reader. Recorded values for the recombinant pneumolysin were used to plot a curve of OD405 vs ng/ml pneumolysin concentration and test samples were quantified in ng per ml using the equation of the curve.

### Haemolytic assay

In all, 4% red blood cell solution was prepared by adding 400 µl of sheep blood pellet to 10 ml of PBS. Bacterial stocks were thawed, centrifuged and resuspended to 1 × 10^7^CFU per ml. Bacteria were lysed using Penicillin and Streptomycin antibiotics at room temperature for 30 min and Miles and Misra counts were performed to check for 100% lysis (Supplementary Fig. 8). A 1:1 ratio of lysed bacteria to 4% RBC solution was added to a microplate before bacteria was diluted twofold with 4% RBC solution. After 30 min incubation the plate was centrifuged and supernatant was removed from each well and placed in a microplate. The OD was measured at 540 nm on a spectrometer to determine the levels of haemoglobin released.

### Statistics

Statistical analysis was carried out using the GraphPad Prism 6® version 5 statistical package (GraphPad Software, Inc). The statistical significance according to the *P*-values were summarised as follows: *P-value < 0.05, **P-value < 0.01, ***P-value < 0.001 and ****P-value < 0.0001. Reported measurements are from distinct samples and not repeated measures.

### Reporting summary

Further information on research design is available in the Nature Research Reporting Summary linked to this article.

## Supplementary information

Supplementary Information

Reporting Summary

## Data Availability

The source data underlying Figs. 1, 3–9, and Supplementary Figs. 1–5, are provided as a Source Data file.

## Source Data

Source Data
