# Supplementary material for: Increased pathogenicity of pneumococcal serotype 1 is driven by rapid autolysis and release of pneumolysin
Source: Nat Commun. 2020 Apr 20;11:1892. doi: 10.1038/s41467-020-15751-6 (PMC7170840; doi:10.1038/s41467-020-15751-6)
Supplement: Supplementary file 1 — Supplementary Information [file 41467_2020_15751_MOESM1_ESM.pdf]

Increased pathogenicity of pneumococcal serotype 1 is driven by rapid autolysis and release of pneumolysin

Supplementary Information

Jacques LC et al.

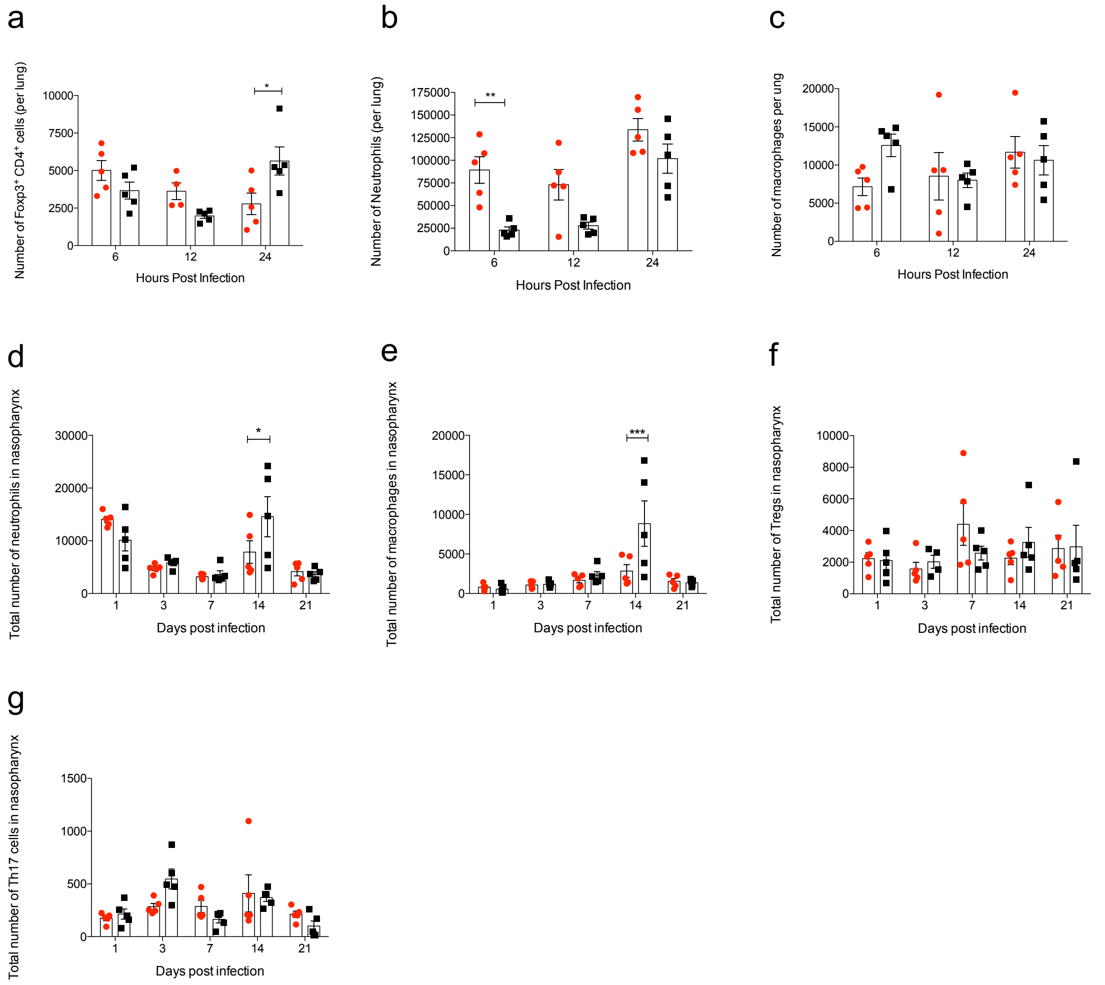

Supplementary Figure 1: **Changes in immune cell numbers in the lung and nasopharynx during serotype 1 (ST217) and serotype 2 (D39) infection.** Infections described previously in Figures 1 and 3. At pre-determined timepoints, mice were culled and the lung (a-c) or nasopharynx (d-g) was processed to create a single cell suspension. Each symbol represents an individual mouse and error bars shown the mean ± SEM. n = 5 mice/group/timepoint. Statistical analysis was performed using Two-way ANOVAs and Sidaks multiple comparisons test. (a) immune cells were stained for CD45 (FITC), CD4 (PE-CY7), CD3 (APC) and Foxp3 (PE) markers to isolate Foxp3^{+} T regulatory cells in the lung. 1(ST217S) infected mice shown in red dots and 2 (D39) infected mice shown in black dots. * P value = 0.0131 (b) cells were stained for CD45 (FITC) and GR1 (PerCP-Cy7) markers to isolate neutrophils in the lung. 1(ST217S) infected mice shown in red dots and 2 (D39) infected mice shown in black dots. ** P value = 0.0030. (c) cells were stained for CD45 (FITC), F4/80 (APC) and CD11b (PE) markers to isolate macrophages in the lung (d) cells were stained for CD45 (FITC) and GR1 (PerCP-Cy7) markers to isolate neutrophils from the nasopharynx. 1(ST217S) infected mice shown in red dots and 2 (D39) infected mice shown in black dots. * P value = 0.0253 (e) cells were stained for CD45 (FITC), F4/80 (APC) and CD11b (PE) markers to isolate macrophages from nasopharynx. 1(ST217S) infected mice shown in red dots and 2 (D39) infected mice shown in black dots. *** P value = 0.0005. (f) Cells were stained for CD45 (FITC), CD4 (PerCP-CY7), Foxp3 (PE) and TGFb (APC) markers to isolate Foxp3^{+} T regulatory cells. 1(ST217S) infected mice shown in red dots and 2 (D39) infected mice shown in black dots (g) Cells were stained for CD45 (FITC), CD4 (PerCP-CY7), RORγT (PE) and IL-17A (APC) markers to isolate Th17^{+} CD4^{+} cells. 1(ST217S) infected mice shown in red dots and 2 (D39) infected mice shown in black dots Source data are provided as a Source Data file.

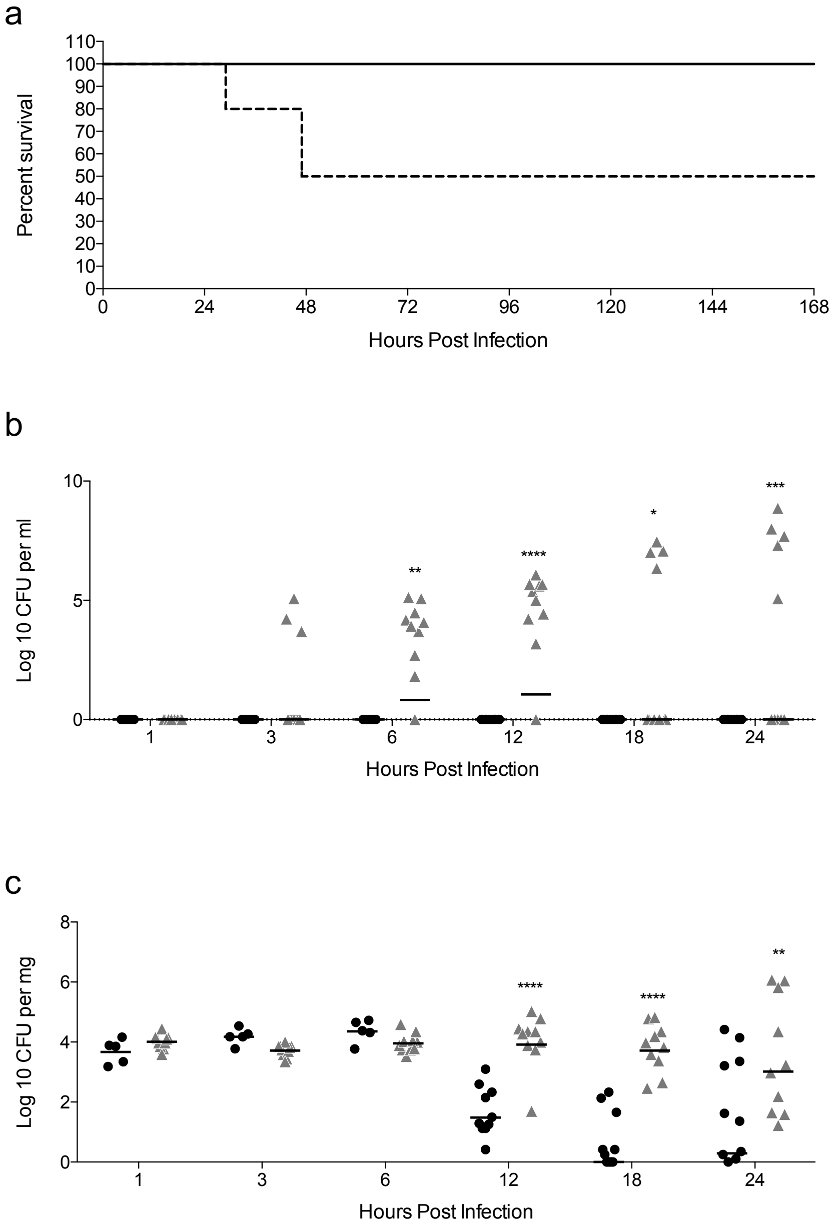

Supplementary Figure 2: Comparison of Serotype 2 (D39) and Serotype 2 expressing ST217 pneumolysin (D39:ST217ply) in BALB/c mouse pneumonia model. (a) Kaplan Meier survival curve showing survival times of mice infected intranasally (IN) with 1 x 10^6 CFU of serotype 2 (D39) (black line) or serotype 2 expressing the ST217 ply allele (D39:ST217ply) (dashed line). b) Blood bacterial load at 1,3, 6, 12, 18 and 24 hours post intranasal infection (CFU per ml). Each dot represents one mouse infected with either D39:ST217ply (grey triangles) or 2(D39)(black circles) and geometric mean shown by horizontal bars. Statistical analysis was performed using Two-way ANOVA and Sidaks's post-test. *P.value = 0.0115, **P.value = 0.0087, ***P.value = 0.0003 and ****P-value<0.0001. (c) Lung bacterial load (CFU/mg) at 1, 3, 6 12, 18 and 24 hours post intranasal infection (each dot represents one mouse). Data are presented as follows; each symbol represents an individual mouse and geometric mean is shown by horizontal bars. Mice infected with D39:ST217ply (grey triangles) or 2(D39)(black circles). Statistical analysis was performed using Two-way ANOVA and Sidaks's post-test. **P.value= 0.0024 and ****P-value<0.0001. Source data are provided as a Source Data file

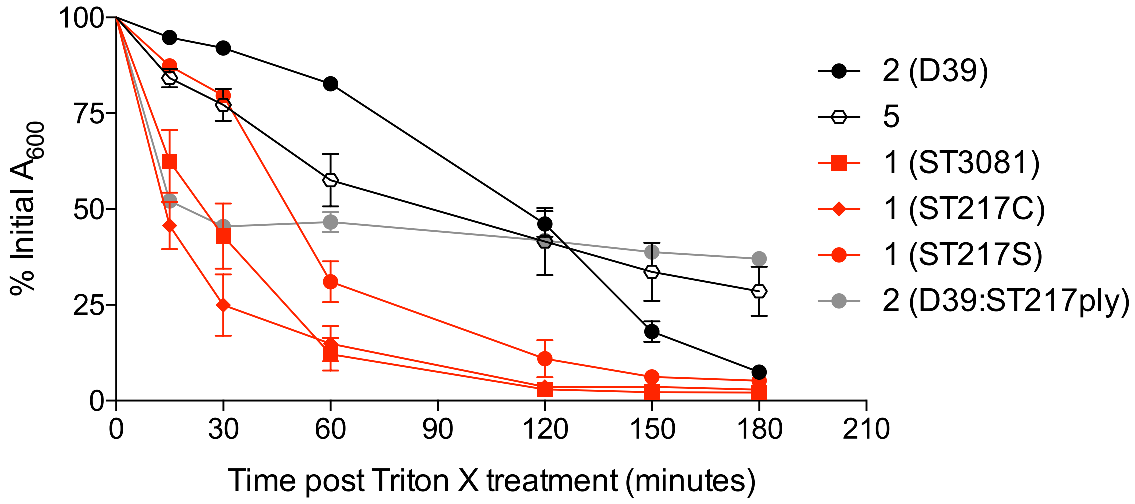

Supplementary Figure 3: Triton X-100-induced autolysis assays show rates of autolysis are significantly higher in serotype 1 isolates compared to serotypes 2 (D39) and 5. Bacteria (OD_{600} 1.0) were incubated at 37^{0}C and 175rpm with 0.01% Triton X. At 15 - 30 minute intervals post treatment, OD_{600} was measured and converted to a percentage of the original OD_{600} reading

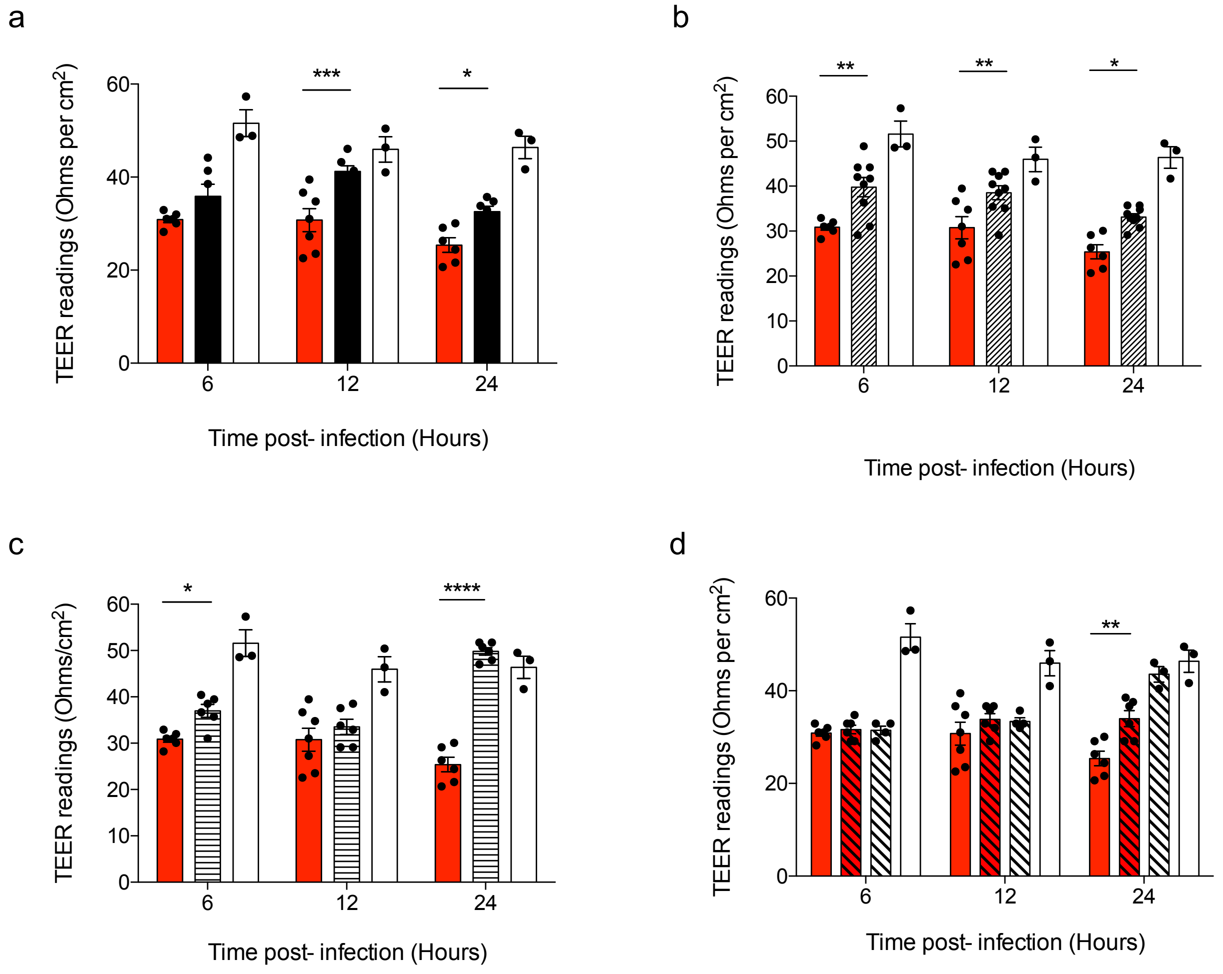

Supplementary Figure 4: African Serotype 1 causes significant damage to epithelial cell barriers. A549 human lung epithelial cells were cultured on trans-well inserts for three days to establish a monolayer. 10^5 cfu of S. pneumoniae were added and trans-epithelial electrical resistance readings (TEER) were taken at 6,12 and 24 hours post infection to assess the damage to tight junctions between epithelial cells. Each dot represents TEER readings from one well and error bars represent the mean ± SEM. Experiment was performed in triplicate. White bars represent control (uninfected) wells of A549 cells. Statistical analysis was performed using Two-way ANOVA and Sidak's multiple comparison test (a) Comparison between serotype 1 (ST217S) (red bars) and serotype 2 (D39) infection (black bars) *P-Value = 0.0280, and ***P-value = 0.0007 (b) Comparison between serotype 5 (diagonal hashed bars) and serotype 1 (ST217S) (red bars). 6 hours **P-Value = 0.0028, 12 hours **P-Value = 0.0065, 24 hours *P-Value = 0.0102 (c) Comparison between serotype 1 (ST306) (horizon striped bars) and serotype 1 (ST217S) (red bars). *P-Value = 0.0396 and ****P value <0.0001. (d) Comparison between serotype 1 (ST217S) (red bars) and serotype 1 (ST217S) + Liposomes (red diagonally hashed bars). Clear, diagonally hashed bars represent A549 cells treated with liposomes only. **P-Value = 0.0026.

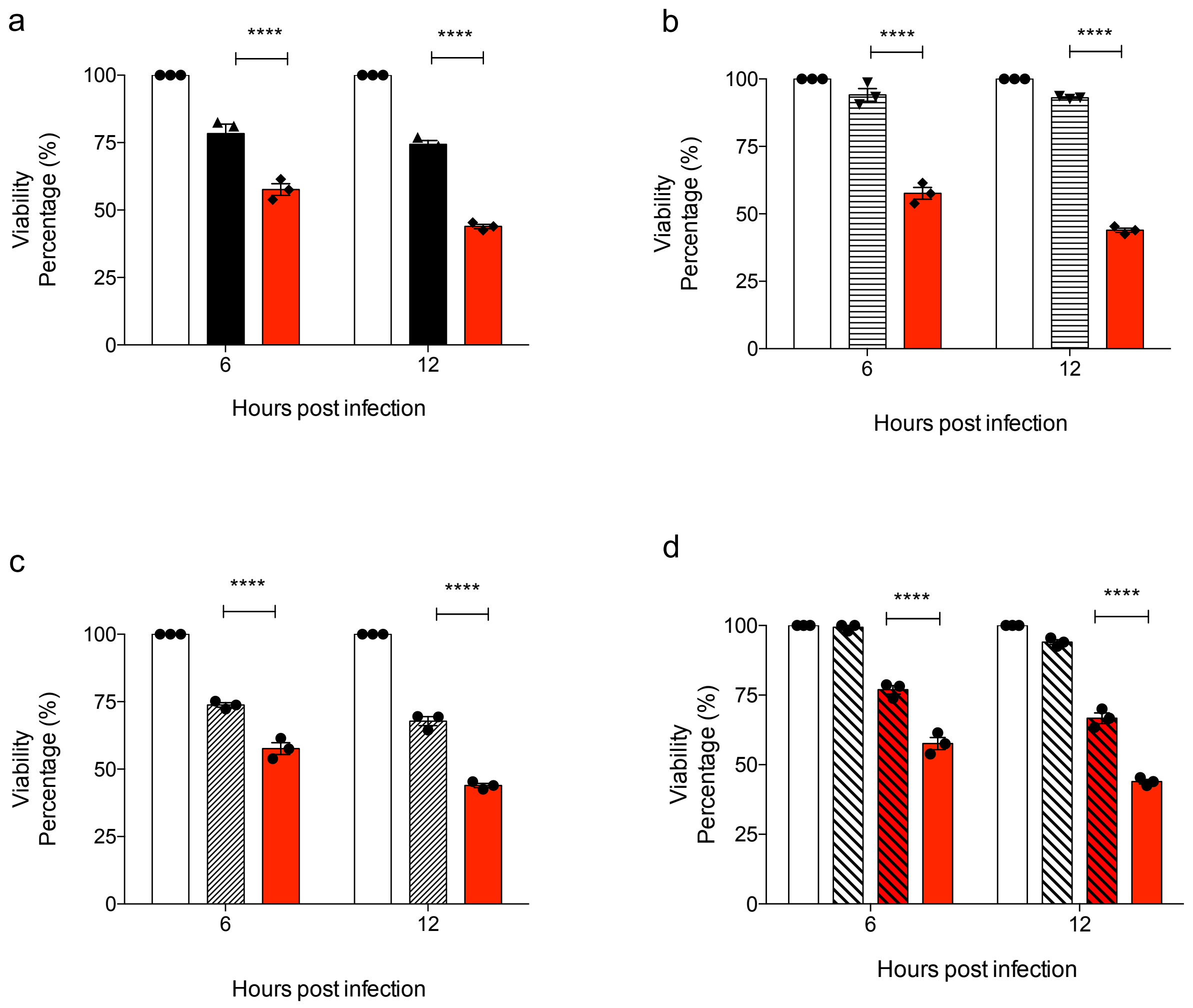

Supplementary Figure 5: Infection with ST217 causes significant decreases in cell viability compared to other serotypes of S. pneumoniae. MTT assay was used to measure cell viability of infected primary human pulmonary epithelial cells after 6 and 12 hours post infection with different serotypes of S. pneumoniae. Experiments were performed in triplicate and data presented with mean ± SEM. Statistical analysis was performed using Two-way ANOVA and Tukey's multiple comparisons test. **** P value <0.0001. (a) compares percentage viability of cells infected with 1 (ST217S) (red bars) and 2 (D39) infected cells (balck bars). (b) compares percentage viability of cells infected with 1 (ST217S) (red bars) and 1 (ST306) infected cells (horizontal striped bars). (c) compares percentage viability of cells infected with 1 (ST217S) (red bars) and serotype 5 infected cells (diagonal hashed bars). (d) compares percentage viability of cells infected with 1 (ST217S) (red bars) and cells infected with 1 (ST217S) with addition of liposomes (red diagonally hashed bars). Cells were also treated with liposomes alone (clear diagonally hashed bars). Viability of non-infected cells are shown (white bars) in S5 (a-d).

Sequencing of ply gene in Serotype 1 (ST217) and Serotype 1 (D39)

Analysis of the upstream region:
Nucleotide position: 1611, **base A** (ST217) > **base G** (S2 D39).

The putative -35 and -10 promoter sequences and the putative Shine-Dalgarno sequence are the same for the two isolates.

Analysis of the downstream region:

Nucleotide positions have been determined according to the sequence of the PCR product upstream + ply gene + downstream sequences = 3428 bp.

	Mutations/Polymorphism	
Nucleotide position	Serotype 1 (ST217)	Serotype 2 (D39)
3368	T	G
3478	A	G
3491	A	G
3703	T	C
3715	G	A
3738	C	A
3757	G	T
3823	T	C
3909	G	A

Analysis of the ply gene according to the gene sequence from the ATG (1) to the stop codon (472):

AA Position	92	153	186	205	346	465
Serotype 1 (ST217)	GTC	ATC	GGC	GAT	AAT	GAA
Coded AA	Val	Ile	Glu	Asp	Asn	Glu
Serotype 2 (D39)	GTT	ATA	GGT	GAC	AAC	GAG
Coded AA	Val	Ile	Glu	Asp	Asn	Glu

Supplementary Figure 6: Sequence analysis of serotype 1 (ST217) and serotype 2 (D39).

	Free Energy (kcal/mol)	Ensemble diversity
**D39V ply**	-423.41	282.99
**Serotype 1 ply**	-421.61	353.55

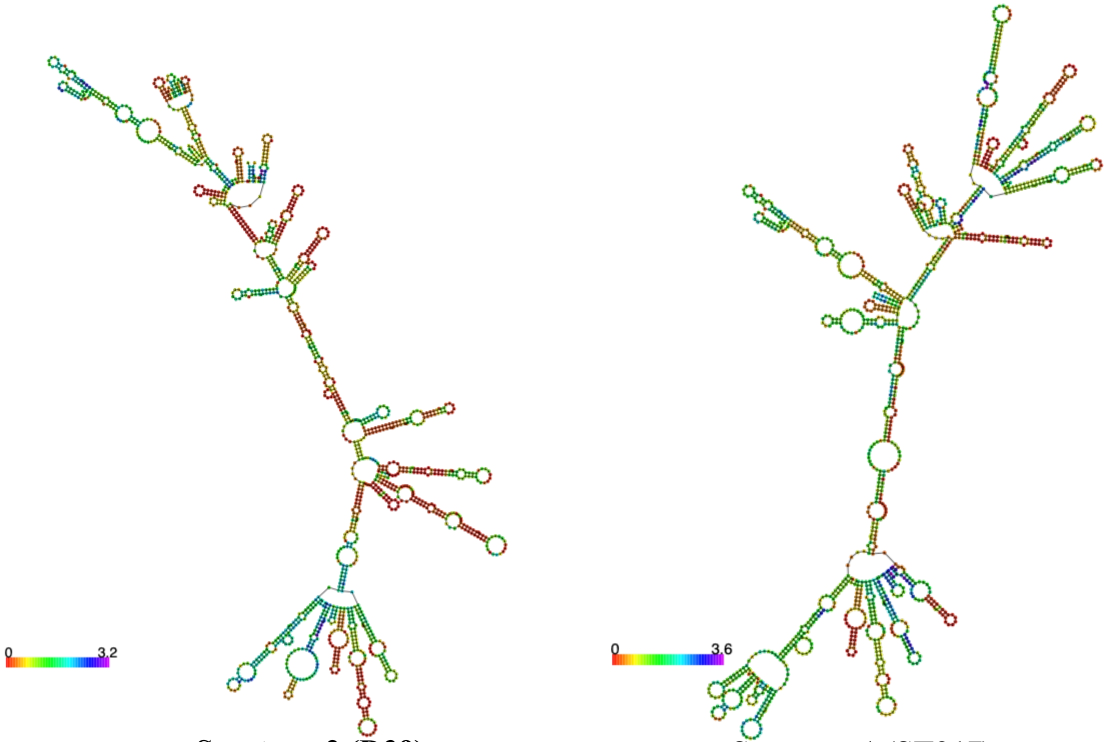

Serotype 2 (D39) Pneumolysin

Serotype 1 (ST217) Pneumolysin

Supplementary Figure 7: **Predicted mRNA structures of ST217 pneumolysin and D39 pneumolysin genes.** RNAfold Web software was used to predict the secondary structure of ST217 pneumolysin compared to D39 pneumolysin, based on 6 single nucleotide polymorphisms (SNPs) in the pneumolysin sequence data.

A

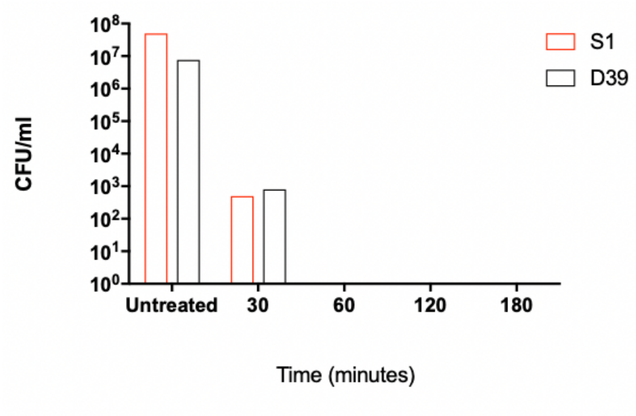

B

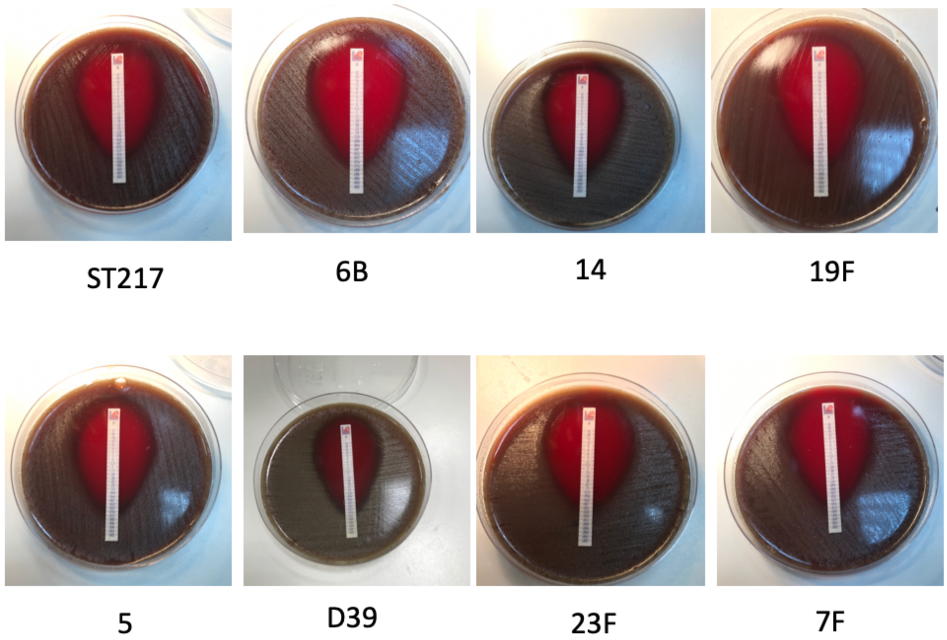

Supplementary Figure 8: All isolates used in the study show sensitivity to penicillin. (A) – CFU counts were performed on 2(D39) and Serotype 1 (ST217S) after treatment with a combination of penicillin and streptomycin treatment. Experiment was performed on three separate cultures per serotype. 100% lysis was achieved 60 minutes post antibiotic incubation. (B) – Different serotypes of S. pneumoniae were cultured overnight on BAB plates with penicillin strips to confirm antibiotic sensitivity.

Foxp3 Treg gating strategy for lung

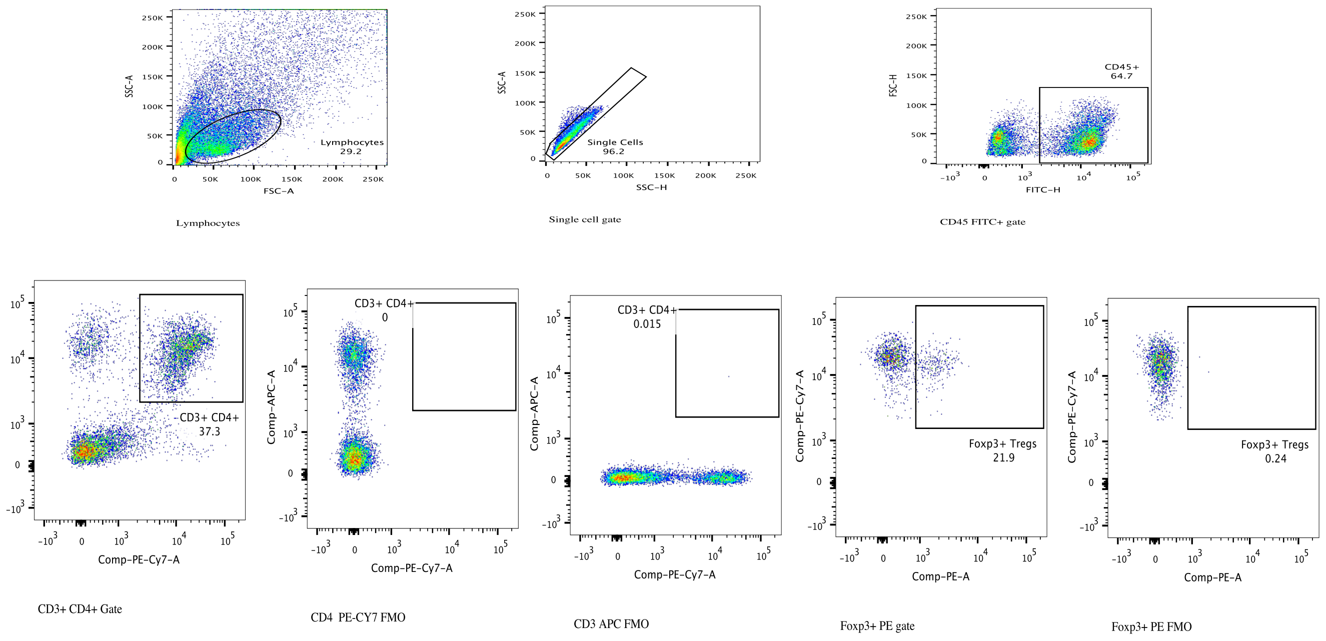

Supplementary figure 9: Gating strategy for murine lung Foxp3^{+} T regulatory cells. Moving from left to right, firstly, lymphocytes were gated according to their forward and side scatter properties. Then single cells were gated on to remove doublets. CD45^{+} positive cells single cells were then gated. Next, cells that were double positive for CD4 and CD3 were selected and Foxp3^{+} expression measured. Fluorescence minus one (FMO) were used as negative controls.

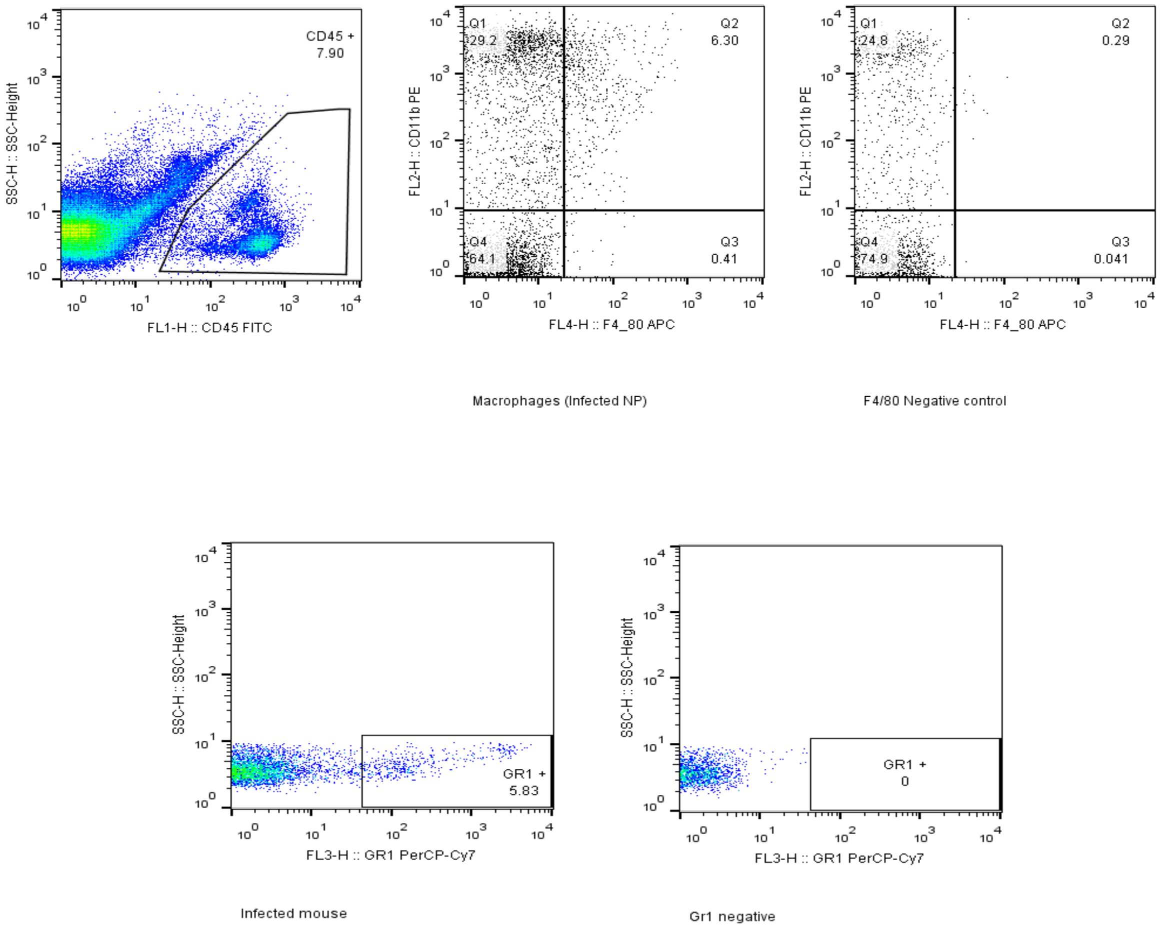

Supplementary figure 10: Gating strategy for murine lung and nasopharyngeal macrophages and neutrophils. CD45 positive cells were isolated and those that we double positive for CD11b and F4/80 were isolated as macrophages. CD45+ GR1+ cells were gated on for neutrophil numbers. Fluorescence minus one (FMO) were used as negative controls.

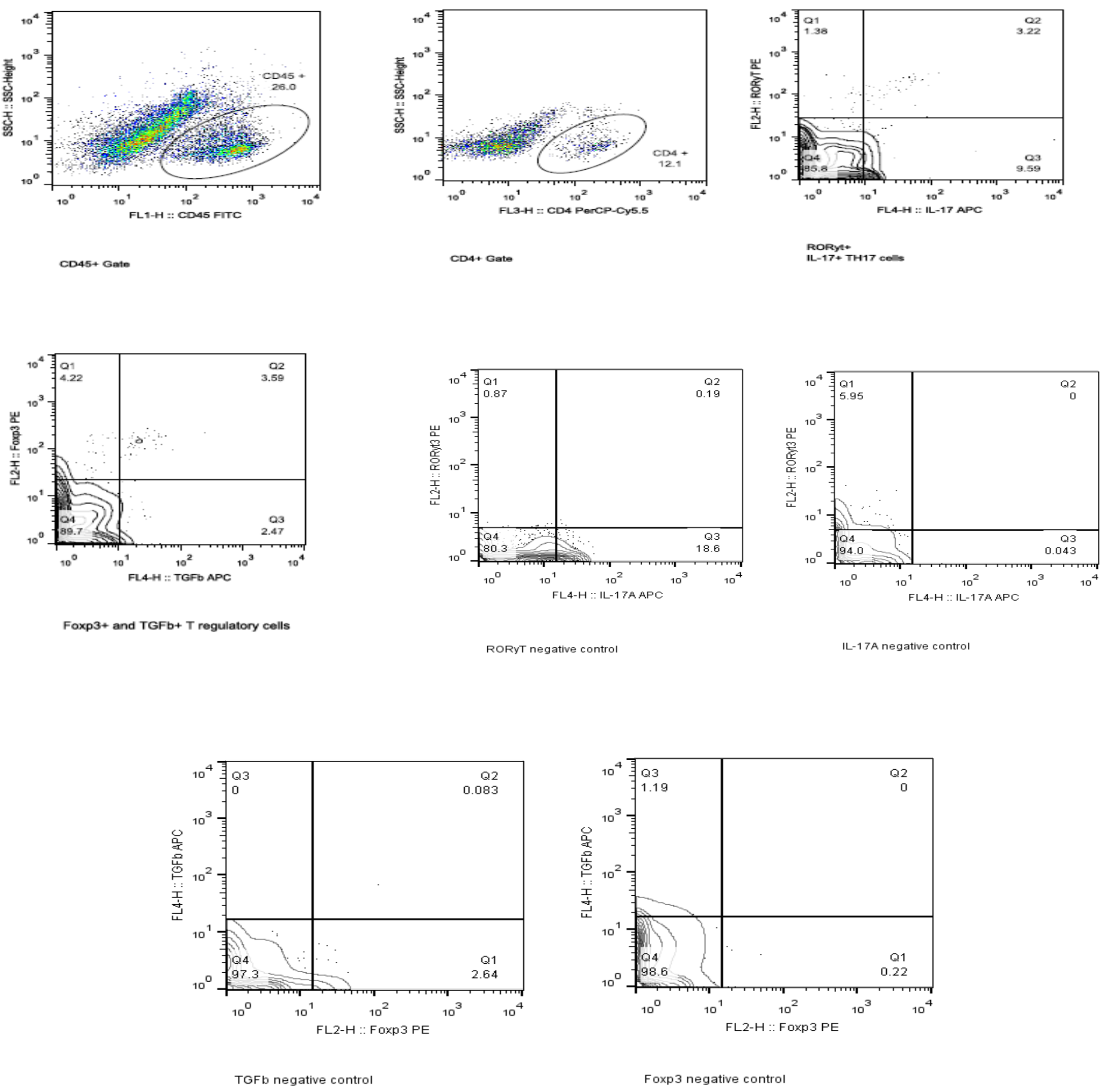

Supplementary figure 11: Gating strategy for murine nasopharyngeal Foxp3$^+$ T regulatory cells. Moving from left to right, firstly, CD45$^+$ positive cells single cells were gated. Next, cells that were positive for CD4 were selected. Th17 cells were defined as IL-17A and RORyT double positive. T regulatory cells were defined as Foxp3 and TGFb double positive. Fluorescence minus one (FMO) were used as negative controls.

SNP	Folding Window	Local region	distance	p-value
U276C	76-476	151-288	0.0080	0.8319
A459C	259-659	429-478	0.0488	0.4400
U558C	358-758	473-644	0.0165	0.6994
C615U	415-815	578-627	0.0537	0.3969
C1038U	838-1238	1020-1069	0.0963	0.2362
G1395A	1195-1416	1358-1408	0.0761	0.2833

Supplementary table 1; showing the predicted effect of SNPs on the overall structure of the mRNA for the ply gene.

Serotype	Type/Source
2 (D39)	D39 Laboratory (NCTC 7466)
5	Clinical isolate (Switzerland)
6B	Clinical isolate, nasopharynx (Switzerland)
19A	Clinical isolate, human (Hungary)
23F	Clinical isolate, nasopharynx (Switzerland)
7F	208.41 Clinical isolate, nasopharynx (Switzerland)
14	Clinical isolate, blood (Switzerland)
19F	ST162 Clinical carriage isolate
PLN-A	Pneumolysin negative serotype 2 D39 mutant,
$LytA^-$	LytA (autolysin) negative serotype 2 D39 mutant,
1 (ST306)	Empyema isolate, Switzerland.
1 (ST217S)	ST217 Clinical isolate from blood (Malawi)
1 (ST217C)	ST217 Clinical isolate from the nasopharynx (Malawi)
1 (ST3081)	ST3081 invasive clinical isolate (Gambia)

Supplementary table 2: Summary of S. pneumoniae serotypes used in study.

Antibody panels used:

Target<br>Cell/protein	Supplier	Antibodies used<br>(catalogue no./lot)	Clone	Dilution	Application<br>(Quality tested?)	Literature matching application
**Neutrophils**	eBioscience®	CD45 FITC (48-0451-82/E00305-1634)	30-F11	1 in 200	FACS (Yes)	NPJ Vaccines. 2017 Jan 23;2:1. doi: 10.1038/s41541-016-0001-5. eCollection 2017
	BioLegend®	Gr-1 PerCP-Cy7*(108416/B231188)	RB6-8CS	1 in 600	FACS (Yes)	Dzhagalov I, et al. 2007. Blood 109:1620. (FC)
**Macrophages**	eBioscience®	CD45 FITC**	30-F11	1 in 200	FACS (Yes)	**
	BioLegend®	F4/80 APC (123116/B268075)	BM8	1 in 400	FACS (Yes)	Poeckel et al. 2009 J. Biol Chem.284:21077
	eBioscience®	CD11b PE(17-0112-82)	M1/70	1 in 600	FACS (Yes)	Front Immunol. 2016 Dec 12;7:597. doi: 10.3389/fimmu.2016.00597. eCollection 2016
**T regulatory cells**	BioLegend®	CD4 PE-Cy7(100528/B258628)	G.K 1.5	1 in 400	FACS (Yes)	León-Ponte M, et al. 2007. Blood 109:3139. (FC)
	eBioscience®	CD45 FITC**	30-F11	1 in 200	FACS (Yes)	**
	eBioscience®	FoxP3 PE(12-4771-82)	NRRF-30	1 in 400	FACS (Yes)	Front Immunol 2018 Aug 22;9:1914. Doi:10.3389/fi,,u.2018.01914.eCollection 2018.
	BioLegend®	TGFβ APC(141406/B219400)	TW7-16B4	1 in 400	FACS (Yes)	Oida T, et al. 2010. PLoS One 5:e15523. (FC, IP, WB)
**Th17 cells**	BioLegend®	CD4 PE-CY7**	G.K 1.5	1 in 400	FACS (Yes)	**
	eBioscience®	CD45 FITC**	30-F11	1 in 200	FACS (Yes)	**
	eBioscience®	RORγT PE(12-6988-82)	AFKJS-9	1 in 400	FACS (Yes)	Wellcome Open Res 2017 Dec 14;2:117.doi:10.12688/wellcomeopenres.13199.3. eCollection2017
	eBioscience®	IL-17A APC(17-7177-81)	eBio17B7	1 in 400	FACS (Yes)	Front Pharmacol. 2018 Jan 9;8:959. doi: 10.3389/fphar.2017.00959. eCollection 2017
**HPAEpiCs**	Abcam®	ZO1(ab96587/GR4665202-1)	Amino acids 1 – 266 of Human ZO1 tight junction protein	1 in 100	IHC(Yes)	Cell Prolif N/A:e12547(2-10)
	Abcam®	DyLight® 488(ab96883/GR5454962-1)	Polyclonal	1 in 1000	IHC(Yes)	PloS One 13:e0193196(2018)
**Pneumolysin**	Abcam®	PLY(ab71810/GR3246176-1)	PLY-4	1µg/well	ELISA(No) In-house setup	PLoSPathog13e:1006582(2017)
	Abcam®	PLY(ab71811/GR3183111-2)	Polyclonal	1µg/well	ELISA(No) In-house setup	Nat Microbiol4:62-70(2019)

Supplementary table 3. Antibodies and dilutions used for FACS, IHC and ELISA analysis of different cell types. **same antibody as previously
