# Supplementary material for: Increased pathogenicity of pneumococcal serotype 1 is driven by rapid autolysis and release of pneumolysin
Source: Nat Commun. 2020 Apr 20;11:1892. doi: 10.1038/s41467-020-15751-6 (PMC7170840; doi:10.1038/s41467-020-15751-6)
Supplement: Supplementary file 2 — Reporting Summary [file 41467_2020_15751_MOESM2_ESM.pdf]

nature research

Corresponding author(s): Aras Kadioglu

Last updated by author(s): Mar 6, 2020

Reporting Summary

Nature Research wishes to improve the reproducibility of the work that we publish. This form provides structure for consistency and transparency in reporting. For further information on Nature Research policies, see Authors & Referees and the Editorial Policy Checklist .

Statistics

For all statistical analyses, confirm that the following items are present in the figure legend, table legend, main text, or Methods section.

n/a	Confirmed	
☐	☒	The exact sample size (n) for each experimental group/condition, given as a discrete number and unit of measurement
☐	☒	A statement on whether measurements were taken from distinct samples or whether the same sample was measured repeatedly
☐	☒	The statistical test(s) used AND whether they are one- or two-sided<br>*Only common tests should be described solely by name; describe more complex techniques in the Methods section.*
☒	☐	A description of all covariates tested
☒	☐	A description of any assumptions or corrections, such as tests of normality and adjustment for multiple comparisons
☐	☒	A full description of the statistical parameters including central tendency (e.g. means) or other basic estimates (e.g. regression coefficient) AND variation (e.g. standard deviation) or associated estimates of uncertainty (e.g. confidence intervals)
☐	☒	For null hypothesis testing, the test statistic (e.g. F, t, r) with confidence intervals, effect sizes, degrees of freedom and P value noted<br>*Give P values as exact values whenever suitable.*
☒	☐	For Bayesian analysis, information on the choice of priors and Markov chain Monte Carlo settings
☒	☐	For hierarchical and complex designs, identification of the appropriate level for tests and full reporting of outcomes
☒	☐	Estimates of effect sizes (e.g. Cohen's d, Pearson's r), indicating how they were calculated

Our web collection on statistics for biologists contains articles on many of the points above.

Software and code

Policy information about availability of computer code

Data collection	BD FACSDiva™ v8.0 for BD FACSCanto™ Flow Cytometers, Omega series V1.51, SkanIt (Research Edition) for Multiskan Spectrum 2.2 software,
Data analysis	Prism 8 for MacOs version 8.4.0/455, ImageJWin64 version 2.0.0-rc-69/1.52p, FlowJo™ v10.6.2

For manuscripts utilizing custom algorithms or software that are central to the research but not yet described in published literature, software must be made available to editors/reviewers. We strongly encourage code deposition in a community repository (e.g. GitHub). See the Nature Research guidelines for submitting code & software for further information.

Data

Policy information about availability of data

All manuscripts must include a data availability statement. This statement should provide the following information, where applicable:
- Accession codes, unique identifiers, or web links for publicly available datasets
- A list of figures that have associated raw data
- A description of any restrictions on data availability

All relevant data are available from the authors

Field-specific reporting

Please select the one below that is the best fit for your research. If you are not sure, read the appropriate sections before making your selection.

☒ Life sciences ☐ Behavioural & social sciences ☐ Ecological, evolutionary & environmental sciences

Life sciences study design

All studies must disclose on these points even when the disclosure is negative.

Sample size

Initial mouse experiments were powered to detect a >25% difference in mean survival between serotype 2 and serotype 1 infected animals. Survival is typically 100% in serotype 2 infected BALB/c animals (Neill DR et al, PLoS Pathogens, 2012, e1002660), but from previous experiments we know that unexpected death occurs in approximately 5% of cases, where co-morbidities are present. From previous work, we know that more virulent serotypes typically have greater variation in survival proportions (standard deviation calculated as 32%) (Bricio-Moreno L, J Infect Dis 2017, 216(10):1318-1327). Thus, using the methodology described by Shah (Shah H, Natl J Physiol Pharm Pharmacol., 2011) for sample size calculations for dichotomous variables (time to death), detecting a 25% difference in means with a standard deviation of 5% in the control group and 32% in the test group would require 10 mice per group (90% power, alpha 0.05). Sample sizes for time point studies were initially chosen on a pragmatic basis, informed by previous experience of detecting differences in colonisation density over time in nasopharynx (Neill DR et al, Am J Respir Crit Care Med 2014, 189 (10):1250-1259) and lungs and blood (Neill DR et al, PLoS Pathogens, 2012, e1002660). Initial experiments looking at lung and blood bacterial burdens in serotype 2 and serotype 1 infected mice, with 5 mice per group, suggested no difference between groups prior to 12 hours post-infection but a 129% difference thereafter (Log10 1.33 vs 3.05, std dev 0.69 vs 1.4). On this basis, we calculated that a total sample size of 9 mice per group would be needed for 90% power, alpha 0.05, using the methodology described above. We included an additional mouse at each time point to account for potential loss due to comorbidities or premature progression to licence severity endpoints.

Data exclusions

No data was excluded

Replication

Data in the following figures are from three independent experiments: Figure 1A, Figure 4C, Figure 5A, Figure 6, Figure 7A-D, Figure 9A-B, Supp Fig 3, Supp Fig 4.

Data in the following figures are from two independent experiments: Figure 1B and C (12-24 hour data), Figure 4B, Supp Fig 5.

Data in the following figure are from a single experiment: Figure 1B and C (1-6 hour data), Figure 2, Figure 3, Figure 4A, Figure 5B, Figure 8A-D, Supp Figure 1A-H, Supp Figure 2, Supp Figure 8.

Randomization

Mice were randomized to cages on arrival at the University of Liverpool animal facility, by technical staff with no role in study design. At study onset, random allocation of unique cage I.D. numbers to experimental groups was performed by the research team. All mice used were female BALB/c of 6-10 weeks of age. Within each experiment, all mice used were within 2 weeks of age of each other.

Blinding

No blinding was performed.

Reporting for specific materials, systems and methods

We require information from authors about some types of materials, experimental systems and methods used in many studies. Here, indicate whether each material, system or method listed is relevant to your study. If you are not sure if a list item applies to your research, read the appropriate section before selecting a response.

Materials & experimental systems

n/a	Involved in the study
☐	☒ Antibodies
☐	☒ Eukaryotic cell lines
☒	☐ Palaeontology
☐	☒ Animals and other organisms
☒	☐ Human research participants
☒	☐ Clinical data

Methods

n/a	Involved in the study
☒	☐ ChIP-seq
☐	☒ Flow cytometry
☒	☐ MRI-based neuroimaging

Antibodies

Antibodies used

Target Cell/protein
Supplier Antibodies used(catalogue no./lot)
Clone
Dilution
Application (Quality tested?)
Literature matching application

Neutrophils

eBioscience
CD45 FITC (48-0451-82/E00305-1634)

30-F11
1 in 200
FACS (Yes)
NPJ Vaccines. 2017 Jan 23;2:1. doi: 10.1038/s41541-016-0001-5. eCollection 2017

BioLegend
Gr-1 PerCP-Cy7*(108416/B231188)
RB6-8CS
1 in 600
FACS (Yes)
Dzhagalov I, et al. 2007. Blood 109:1620. (FC)

Macrophages

eBioscience
CD45 FITC**
30-F11
1 in 200
FACS (Yes)
**
BioLegend
F4/80 APC (123116/B268075)
BM8
1 in 400
FACS (Yes)
Poeckel et al. 2009 J. Biol Chem.284:21077

eBioscience
CD11b PE(17-0112-82)
M1/70
1 in 600
FACS (Yes)
Front Immunol. 2016 Dec 12;7:597. doi: 10.3389/fimmu.2016.00597. eCollection 2016

T regulatory cells

BioLegend
CD4 PE-Cy7(100528/B258628)
G.K 1.5
1 in 400
FACS (Yes)
León-Ponte M, et al. 2007. Blood 109:3139. (FC)

eBioscience
CD45 FITC**
30-F11
1 in 200
FACS (Yes)
**
eBioscience
FoxP3 PE(12-4771-82)
NRRF-30
1 in 400
FACS (Yes)
Front Immunol 2018 Aug 22;9:1914. Doi:10.3389/fi,,u.2018.01914.eCollection 2018.

BioLegend
TGFAPC(141406/B219400)
TW7-16B4
1 in 400
FACS (Yes)
Oida T, et al. 2010. PLoS One 5:e15523. (FC, IP, WB)

Th17 cells

BioLegend
CD4 PE-CY7**
G.K 1.5
1 in 400
FACS (Yes)
**
eBioscience
CD45 FITC**
30-F11
1 in 200
FACS (Yes)
**

eBioscience
RORT PE(12-6988-82)
AFKJS-9
1 in 400
FACS (Yes)
Wellcome Open Res 2017 Dec 14;2:117.doi:10.12688/wellcomeopenres.13199.3. eCollection2017

eBioscience
IL-17A APC(17-7177-81)
eBio17B7
1 in 400
FACS (Yes)
Front Pharmacol. 2018 Jan 9;8:959. doi: 10.3389/fphar.2017.00959. eCollection 2017

Mammalian cells (HPAEpiCs)

Abcam ZO1(ab96587/GR4665202-1)
Amino acids 1 – 266 of HumanZO1 tight junction protein
1 in 100
IHC(Yes)
Cell Prolif N/A:e12547(2-10)

Abcam DyLight® 488(ab96883/GR5454962-1)
Polyclonal
1 in 1000
IHC(Yes)
PloS One 13:e0193196(2018)

Pneumolysin
Abcam
PLY(ab71810/GR3246176-1)
PLY-4
1µg/well
ELISA(No)/In-house setup
PLoSPathog13e:1006582(2017)

Abcam
PLY(ab71811/GR3183111-2)
Polyclonal
1µg/well
ELISA(No)/In-house setup
Nat Microbiol4:62-70(2019)

Validation	Validation is shown above in "antibodies used" section but also in a table format in Supplementary data (supplementary table 3).

Eukaryotic cell lines

Policy information about cell lines

Cell line source(s)	A549 human lung carcinoma cell line (acquired from Sigma) and Primary human pulmonary alveolar epithelial cells (HPAEpiC), acquired from ScienCell Research Laboratories – Cat. No 3200/LOT27000
Authentication	A549 cells were authenticated by The European Collection of Authenticated Cell Cultures (ECACC) whereas ScienCell Research Laboratories provide their own quality assurance tests.<br>For both cell lines:<br>CMV,HTLV1,HIV1,HCV,HBV,EBV tested as "Not detected"<br>No endotoxin detected.
Mycoplasma contamination	All cell lines used tested negative for mycoplasma contamination
Commonly misidentified lines<br>(See ICLAC register)	No cell lines used are listed in the database of commonly misidentified cell lines.

Animals and other organisms

Policy information about studies involving animals; ARRIVE guidelines recommended for reporting animal research

Laboratory animals	6-10-week old, female BALB/c were purchased from Harlan Laboratories (Bicester, UK). The animals were housed in the animal facilities under the following conditions:<br>Temperature:21-23 degrees Celsius<br>Humidity:55-65%<br>Cages: Individually ventilated cages (IVC) from Technoplast (GM500)<br>Water supply: Automating watering providing reverse osmosis water sterilised by UV radiation.<br>Enrichment: Nesting material, balcony, dome home, handling tunnel.
Wild animals	Study did not involve wild animals
Field-collected samples	Study did not involve samples collected from the field
Ethics oversight	This study was performed in strict accordance with UK Home Office guidelines. Animal experiments were performed at the University of Liverpool and were approved by the University of Liverpool Animal Welfare Research Body (AWERB).

Note that full information on the approval of the study protocol must also be provided in the manuscript.

Flow Cytometry

Plots

Confirm that:

☒ The axis labels state the marker and fluorochrome used (e.g. CD4-FITC).

☒ The axis scales are clearly visible. Include numbers along axes only for bottom left plot of group (a 'group' is an analysis of identical markers).

☒ All plots are contour plots with outliers or pseudocolor plots.

☒ A numerical value for number of cells or percentage (with statistics) is provided.

Methodology

Sample preparation	BALB/c mouse lung tissue was harvested, weighed, placed into a petri dish and then cut into smaller pieces using a scalpel blade. To help release immune cells via enzymatic digestion, lung tissue it was placed in 1.5ml Eppendorf tubes containing 1ml of PBS and 10mg per ml of Collagenase D (Roche). The Eppendorf tubes were then incubated at 37oC for 30 minutes. After digestion, tissue was passed through a 40m cell strainer (BD Biosciences) and washed with sterile PBS to create a single cell suspension. Cell suspensions were then centrifuged at 400 x g for 5 minutes. The cell pellet was re-suspended in 1x Red blood cell lysis buffer (Sigma) to lyse all red blood cells. The cell suspensions were then centrifuged at 400 x g for 5 minutes and cell pellet re-suspended in cryopreservation media, for storage at -80oC. When needed, aliquots of cells were thawed quickly in the water bath.<br><br>BALB/c mouse nasopharyngeal tissue was harvested and placed into bijou tubes containing 3mls of sterile PBS. The tissue was then mechanically disrupted for ~ 1 minute using a homogenizer (IKA T10). The homogenized tissue was then passed through a 40m pore cell strainer and centrifuged at 400 x g for 5 minutes. The cell pellet was either re-suspended in cryopreservation media or used for FACS analysis on the same day.<br><br>For staining and acquisition, samples were either thawed or used fresh from dissection. Cells were incubated with a 1 in 200

dilution of purified anti- CD16/CD32 Fc blocking antibody (eBiosciences) for 30 minutes at room temperature. Following incubation with blocking antibody, cell surface markers were stained for 30 minutes at room temperature, in the dark. Cells were fixed and permeabilised for 30-60 minutes at room temperature, in the dark, then an intracellular monoclonal antibody panel was used to detect both intracellular cytokines and transcription factors.

Instrument	BD FACSCanto™ Flow Cytometer
Software	BD FACSDiva™ v8.0 for BD FACSCanto™ Flow Cytometers.Data was analysed on FlowJo™ v10.6.2
Cell population abundance	10,000-50,000 CD45 positive cells were collected per mouse, per tissue. The numbers of neutrophils, macrophages, CD4 T cells, Th17 and Treg cells were then analysed and are displayed on the graphs
Gating strategy	Initial gating on FSC (300) and SSC (50) was designed to exclude debris but to include lymphocytes, monocytes and granulocytes. Doublets were excluded by FSC-H vs FSC-A comparison. Gating was next performed on CD45, to exclude non-haematopoietic cells. Within the CD45+ gate, all cell types were differentiated based on antibody staining, with negative and positive populations discriminated based on isotype control or fluorescence-minus one (FMO) staining. Isotype controls were included for all surface markers except CD45, and FMO staining was included for all intracellular markers. Neutrophils were defined as CD45+Gr-1+, macrophages as CD45+CD11b+F4/80+, T cell subsets as CD45+CD3+CD4+ and then Foxp3+TGFb+ (T regulatory cells) or RORgt +IL-17+ (Th17 cells).

☒ Tick this box to confirm that a figure exemplifying the gating strategy is provided in the Supplementary Information.
